# Nanometer-resolution imaging of living bacteria across species and cellular orientations

**DOI:** 10.1038/s41467-026-78975-y

**Published:** 2026-11-09

**Authors:** Anna Scheeder, Joanna Szczepaniak, Yulianna Koziy, Naomi Mara Claro De Oliveira, William Trewby, Renata Kaminska, Jeremy Brown, Colin Kleanthous, Bart W. Hoogenboom

**Affiliations:** 1 London Centre for Nanotechnology, University College London, London, UK; 2 Department of Biochemistry, South Parks Road, University of Oxford, Oxford, UK; 3 UCL Respiratory, Division of Medicine, University College London, London, UK; 4 Department of Physics & Astronomy, University College London, London, UK

**Keywords:** Atomic force microscopy, Bacteriology, Cellular microbiology, Bacterial techniques and applications, Imaging

## Abstract

The bacterial cell envelope is a complex superstructure that is an important target and a major barrier for antibiotics. To date, highest-resolution images of native cell envelopes have been obtained by atomic force microscopy (AFM), but its use is limited by challenges in bacterial immobilization. We establish glutaraldehyde-functionalized glass surfaces for reversible covalent attachment, enabling robust adhesion across species and cellular orientations while preserving cell viability and growth. This approach allowed imaging of *Escherichia coli* both expressing O-antigen and fully lacking lipopolysaccharide (LPS) oligosaccharides, revealing that AFM contrast depends on LPS complexity and confirming previously observed outer membrane domains as LPS-enriched islands. It also enabled imaging of *Pseudomonas aeruginosa* and *Acinetobacter baumannii* outer membranes, which lack the characteristic protein trimers observed in model *E. coli*. Finally, we discovered growth-phase dependent alterations in *E. coli* pole morphology, where trimer networks typical of exponential-phase cells were unresolved and morphological defects appeared.

## Abstract (web summary)

The use of atomic force microscopy for high-resolution imaging of the surface of live bacterial cells is limited by challenges in cell immobilization. Here, Scheeder et al. present an improved immobilization method that allows imaging of diverse bacteria, including imaging of bacterial cell poles.

## Introduction

Most last-resort antibiotics target the bacterial cell envelope, notably the peptidoglycan cell wall and, in Gram-negative bacteria, the outer membrane, which in itself is also a major barrier to many antibiotics^1,2^. Despite the importance of the cell envelope, its molecular architecture has long remained obscure. This is linked to its rather disordered nature, the relative lack of transparency of bacterial cells for electron microscopy, and to the nanometer scale at which this architecture is defined, making it less amenable to optical microscopy approaches^3–5^.

As a complementary technique, atomic force microscopy (AFM) has been successful in revealing molecular and supramolecular architectures in the cell envelopes of Gram-positive and Gram-negative bacteria^4,6–15^. This includes the early visualization of hexagonally ordered surface layers of glycoproteins on *Corynebacterium glutamicum*^16^, as well as, more recently, the amorphous, mesh-like cell wall of *Staphylococcus aureus*, *Bacillus subtilis*, and *Escherichia coli*, which display varying porosity, alignments, and rearrangements of peptidoglycan chains^10,11,17,18^. In the asymmetric proteolipid outer membrane of *E. coli*, AFM has revealed proteinaceous superstructures^7,8,12,13^ and phase separation into imperfectly ordered outer membrane protein networks and lipopolysaccharide (LPS)-rich domains^7^. Moreover, AFM enables the study of bacterial surface dynamics and cell division^19,20^, and provides insights into cell-envelope-based antimicrobial resistance and mechanisms of action of surface-active antibiotics^17,21–23^.

**Fig. 1:**
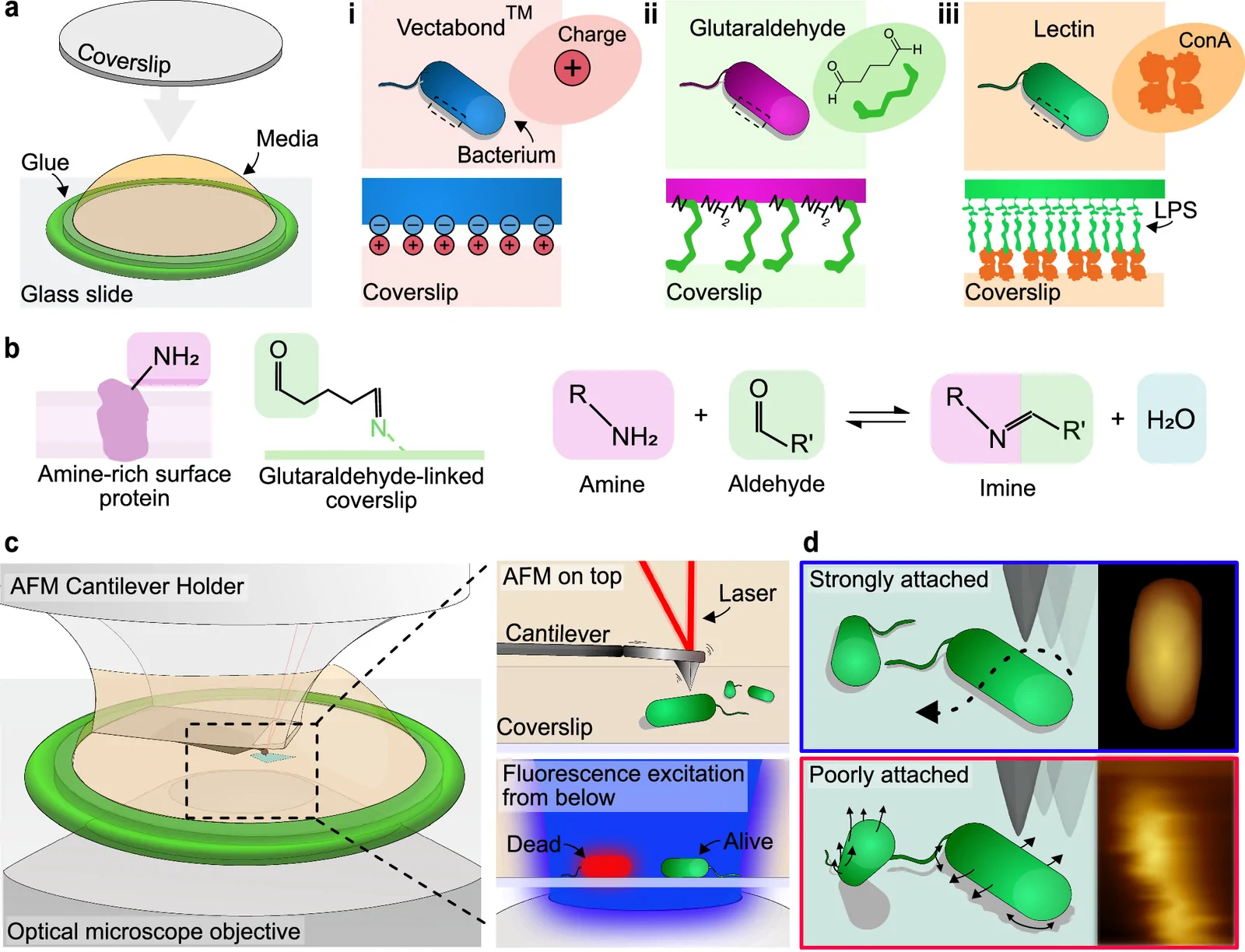
AFM on living bacteria requires robust cell adhesion. **a** Schematic of glass coverslip surface coatings explored for the immobilization of bacteria in this work. (i) Vectabond^TM^ facilitates electrostatic attachment, (ii) glutaraldehyde forms a reversible covalent bond with amines on the bacterial surface, whereas (iii) lectins specifically adhere to sugars of the LPS. Coated coverslips are glued to a glass microscope slide and used for the immobilization of bacteria. **b** Schiff base formation between primary amines of surface lysines and glutaraldehyde-linked glass is reversible in the presence of water, preserving cell viability and the ability to grow and divide while being imaged with AFM. **c** Schematic of correlative AFM and fluorescence microscopy setup. AFM facilitates nanoscale probing of the native cell envelope while an inverted optical microscope is used to locate cells and assess cell viability. **d** Schematic of AFM image output. Inadequate immobilization of bacteria translates into fuzzy and, thereby, at best, low-resolution AFM imaging.

Particularly for live-cell imaging, however, AFM comes with the disadvantage that it relies on stringent immobilization of bacteria to yield a sufficiently stable surface for AFM imaging (Fig. 1), while not affecting cell viability and (potential for) growth. Previously, bacteria have been immobilized by physical trapping in grids or trenches^11,24,25^, by chemical coupling to substrates^12,13,26^, by attachment via hydrophobic interactions^20^, and, most commonly, by electrostatic adhesion onto surfaces coated with gelatin, poly‑L‑lysine, Cell‑Tak™, or Vectabond™^7,8,11,21,22,27–29^. These immobilization strategies target conserved features of bacterial envelopes, including cell shape, negative surface charge, reactive surface groups in proteins and peptides, and surface carbohydrates.

In practice, these strategies for AFM experiments have led to mixed results for cell attachment and viability across strains. This is evidenced, e.g., by the lack of high-resolution AFM data on smooth *E. coli* (*i.e*., expressing full-length O-antigen)^7^ and unsuccessful use of AFM imaging for live *Pseudomonas aeruginosa*^27^.

Therefore, for nanometer-resolution studies using AFM of cell envelopes across a wider range of bacterial species and strains, significant methodological improvement is needed. To achieve such improvement, we explore and compare different immobilization strategies and find that reversible covalent immobilization using glutaraldehyde-functionalized glass yields remarkably robust results. This approach maintains high cell viability while ensuring rigid attachment, enabling high-resolution AFM imaging across different species, strains, and cellular orientations, including bacterial poles.

## Results

### Reversible covalent immobilization facilitates AFM imaging of smooth *E. coli*

**Fig. 2:**
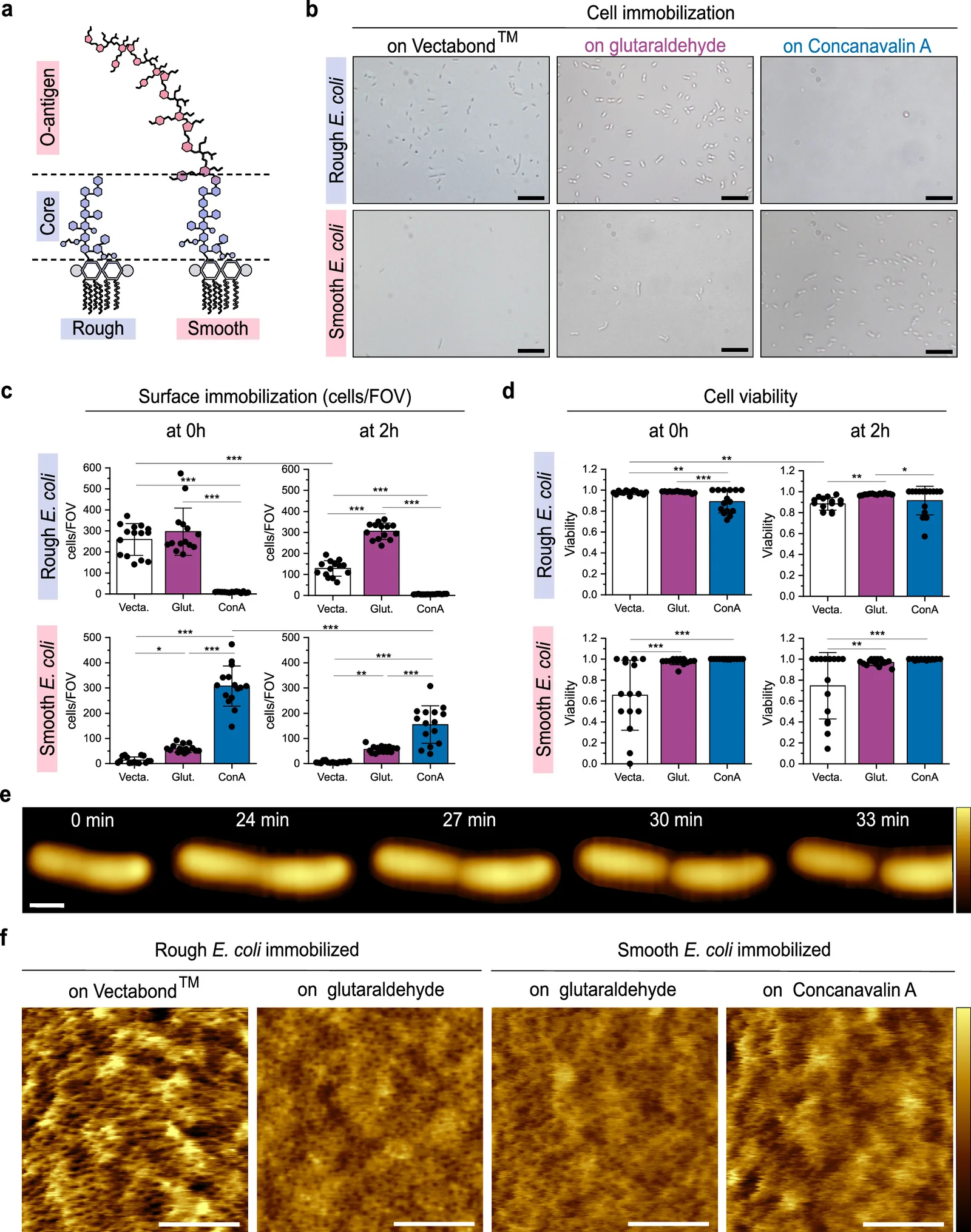
A reversible covalent immobilization strategy enables imaging of living bacterial strains that were previously inaccessible to AFM. **a** Illustration of the LPS of “rough” *E. coli* MG1655 lacking O-antigen and the oligosaccharide extension of the LPS of “smooth” *E. coli* MG1655 with reintroduced O-antigen. **b** Bright-field images of surface-immobilized cells (mid-logarithmic phase) on coated coverslips after repeated surface rinsing. Quantification of surface immobilization as cells/ field of view (FOV), where the FOV measured 237 × 178 µm^2^ (**c**), and cell viability as percentage of cells with maintained cell envelope integrity (negative for SYTOX^TM^) (**d**) of bacteria adhering to the coated coverslips immediately after immobilization (at 0 h) and 2 h after immobilization at room temperature and additional surface rinsing (at 2 h). Data points were acquired from three biological repeats with each five different acquisition areas (*n* = 15, except rough MG1655 on Glut. with *n* = 14). Data are displayed as mean ± s.d. Statistical significance of differences was determined by two-way repeated-measures ANOVA, followed by Holm-Sidak multiple-comparison tests, with significant differences between the means of two groups highlighted with asterisk, *p* < 0.05 = *, *p* < 0.005 = **, *p* < 0.0005 = ***. **e** AFM height image series of a growing (0–24 min) and dividing (27–33 min) *E. coli* MG1655 cell in LB media immobilized on a glutaraldehyde-functionalized coverslip. **f** AFM height images of the bacterial surface of rough and smooth MG1655 in HEPES buffer acquired on the various glass surface coatings. Source data are provided as a Source Data file. Scale bars: 10 µm (**b**); 1000 nm (**e**); 100 nm (**f**). Color (height) scales: 300 nm (**e**); 6 nm (**f**).

The O-antigen polysaccharide sidechain of LPS is a major factor in Gram-negative bacterial antibiotic resistance^30^, but high-resolution AFM imaging has so far been limited to laboratory strains with truncated O-antigen (“rough” strains)^7–9,22,28,31^. This is at least in part because such rough strains are readily immobilized by charge-based functionalization, e.g., on Vectabond^TM^ functionalized glass coverslips^7^. By contrast, when we restored the O-antigen by repairing the *wbbL* mutation of *E. coli* K-12 MG1655 (resulting in a “smooth” strain)^32–34^, adsorption and immobilization were poor, as shown for smooth *E. coli* in Fig. 2a–c.

To facilitate combined AFM and optical microscopy on smooth strains, we therefore explored two alternative strategies to Vectabond^TM^ (Fig. 1a). First, surface-linked glutaraldehyde, as was previously demonstrated for optical imaging of rough *E. coli* on glass^35^ and for AFM of *Magnetospirillum magneticum*, *Rhodobacter sphaeroides* and rough *E. coli* on mica^12,13^. Second, we explored surface-linked lectins as a naturally occurring method to specifically target bacterial surface sugars^36^. The lectin Concanavalin A (ConA) was chosen as a protein with binding specificity for α-D-glucose and α-D-mannose, targeting smooth MG1655 with the restored O-antigen^37,38^, which we verified using fluorescent labelling (Supplementary Fig. 1). The three different functionalization procedures (Vectabond^TM^, glutaraldehyde, and ConA) are discussed in detail in the methods section. The sample preparation time, including glass coverslip functionalization (excluding glass cleaning) and bacterial immobilization prior to AFM imaging, is approximately 15 min for Vectabond^TM^, 1 h for glutaraldehyde functionalization, and 1 h 15 min for ConA-coated slides. No specialized equipment was needed beyond a bath sonicator, plasma cleaner, and fume hood.

To compare surface attachment and cell viability between rough and smooth *E. coli* for the three coverslip functionalizations, initial tests were carried out by optical microscopy. Cells grown to mid-logarithmic phase were prepared to the same optical density (OD = 0.5) in 20 mM HEPES buffer and incubated on treated coverslips. Subsequently, the surfaces were rinsed with minimal medium (MM) to remove unadhered cells. Bright-field images (Fig. 2b) were acquired to count adhered bacteria per field of view (FOV) and thereby quantify the attachment efficiency. With the addition of SYTOX^TM^, the membrane integrity of the bacteria in the same FOV was assessed from fluorescence images (Supplementary Fig. 2) and used as a proxy for cell viability^39^.

As shown and quantified in Fig. 2b–c, the glutaraldehyde functionalization resulted in several tens to hundreds of adhered cells per FOV for both rough and smooth *E. coli*. Similar initial adherence of rough *E. coli* was found on Vectabond^TM^, but glutaraldehyde outperformed Vectabond^TM^ for the immobilization of the smooth strain by a factor **≳** 5. Moreover, in contrast to Vectabond^TM^, glutaraldehyde functionalization also resulted in more durable attachment for both strains as assessed by measuring cell counts after 2 h and an additional washing step. As expected, ConA functionalization yielded high attachment efficiency for the smooth strain and virtually no attachment for the rough strain, as the latter lacks α-D-glucose and α-D-mannose on its surface. While glutaraldehyde binds smooth *E. coli* in abundance, it yielded lower attachment efficiency than for the rough strain on glutaraldehyde and for the smooth strain on ConA-functionalized cover slips. This may be explained by there being fewer accessible amine groups for glutaraldehyde to attach to on the bacterial cell surface with restored O-antigen. Most importantly, cell viability was high (typically at least 90% over 2 h) for all functionalization methods that successfully adhered cells (Fig. 2d), highlighting their suitability for live-cell imaging. For glutaraldehyde, this may at first seem surprising, given its common use as a cell fixative, which could have been expected to compromise viability^40,41^. However, cell fixation relies on crosslinking of primary amine groups, resulting in the rigidification of proteins and other amine-containing molecules. For the bacterial immobilization as described here, glutaraldehyde, with its two aldehyde groups, is on one side covalently linked to the glass substrate, impairing its ability to form cross-links between molecules; any remaining free glutaraldehyde in solution was removed by extensive washing before bacterial incubation. Hence, accessible amines on the bacterial surface only react with free aldehyde groups that are chemically linked to the glass coverslip. Moreover, the Schiff base formation reaction between the bacterium and the functionalized glass results in a covalent bond that is reversible in aqueous solution^42^ (Fig. 1). Cell viability and the dynamic nature of the glutaraldehyde binding were further demonstrated by the growth (0–24 min) and division (27–33 min) of glutaraldehyde-bound bacteria on the surface in the presence of rich media, as illustrated by the AFM image series in Fig. 2e.

Finally, to assess suitability of the different immobilization strategies for high-resolution AFM imaging, we aimed to resolve outer membrane structures reminiscent of protein trimers (OmpF and OmpC), as reported in Fig. 2f. In previous AFM studies of rough *E. coli*, OmpF and OmpC trimers appeared as ~5 nm wide depressions (or apparent pores), in densely packed networks in the outer membrane; this identification was established via an assessment of local lattice parameters, by biochemical labeling and by specific mutations^7^. Vectabond^TM^-immobilized rough *E. coli* facilitated such high-resolution imaging, thereby reproducing earlier OmpF/OmpC observations by Benn et al. (2021)^7^, but AFM imaging of smooth *E. coli* on Vectabond^TM^ remained unsuccessful due to insufficient cell attachment. In contrast, ConA provided sufficient cell attachment for optical imaging (Fig. 2b, c), but the cells were not immobilized rigidly enough for AFM, resulting in lower-resolution images and frequent cell detachment mid-imaging. Only the glutaraldehyde-functionalized cover slips provided sufficiently rigid immobilization to image both rough and smooth *E. coli* at high resolution using AFM.

### Resolving outer membrane protein networks and phase separation as a function of oligosaccharide complexity

Having established an immobilization strategy that enabled high-resolution AFM on bacteria regardless of the polysaccharide, we next used this for an in-depth comparison of the outer membranes of rough and smooth *E. coli*, and of a “clear” *E. coli* strain. Clear *E. coli* completely lacks both the O-antigen and the core oligosaccharide as displayed in Fig. 2a and produces lipid IV(A) with 4 acyl chains, in contrast to the 6 acyl chains of lipid A^43^.

**Fig. 3:**
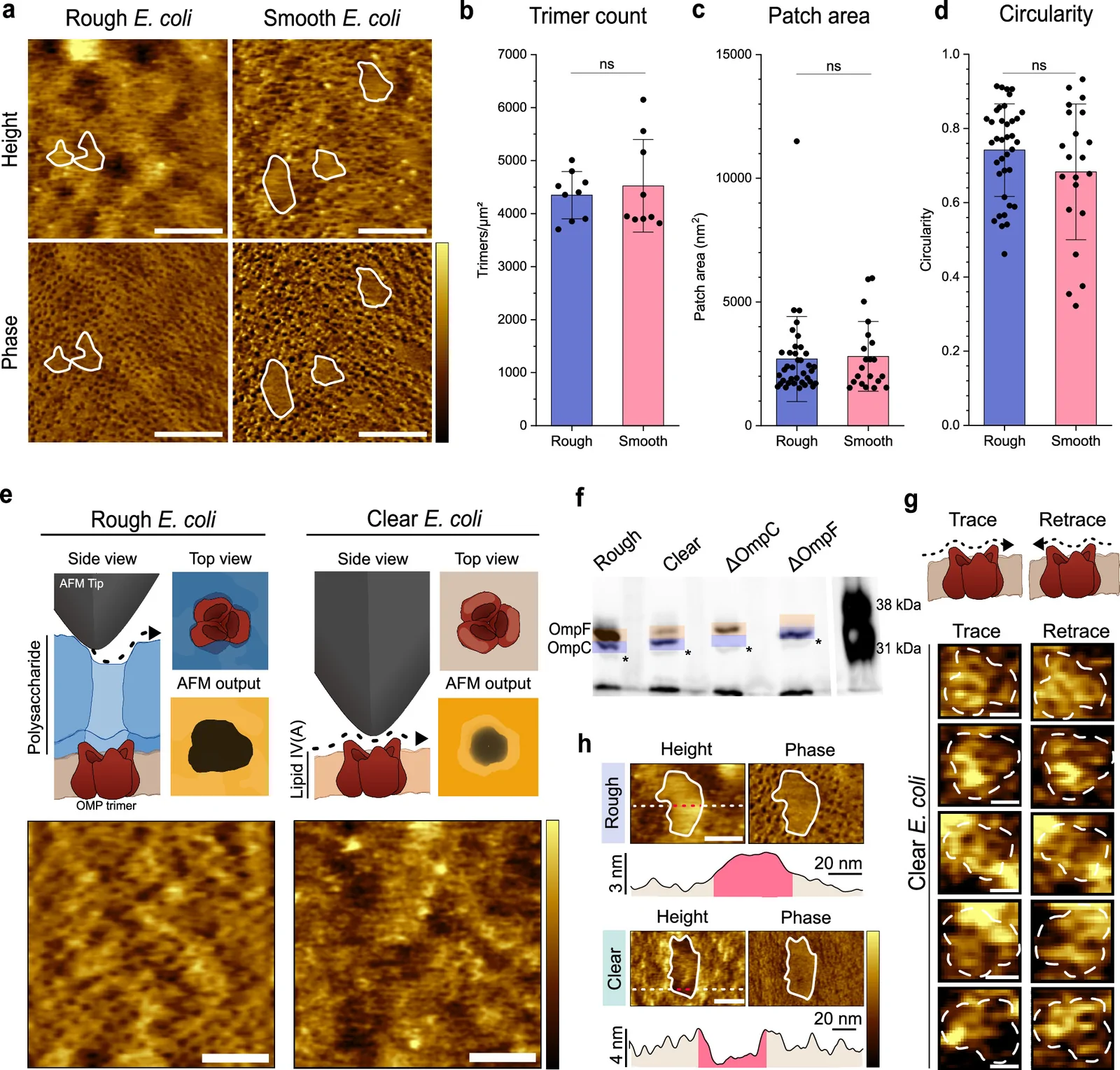
Molecular architecture of the outer membrane with/without polysaccharides. **a** AFM height and phase images of rough and smooth *E. coli* MG1655 cells imaged on glutaraldehyde-coated slides in MM. Regions free of features associated with porin trimers are outlined in white. **b** Porin trimer count per µm^2^, as determined from AFM phase images. Area (**c**) and shape/circularity (**d**) of trimer-free patches as determined from AFM phase images. Data points were acquired from three biological repeats, imaging three cells for each preparation (*n* = 9). Data are displayed as mean ± s.d. Statistical significance of differences was determined by the Mann-Whitney Test. Ns, not significant. **e** Top row: Schematic illustration of AFM imaging of the outer membrane of rough *E. coli* (with polysaccharide) and clear *E. coli* (without oligosaccharide) drawn approximately to scale, with predicted AFM images. Bottom row: Height images recorded for rough and clear MG1655. **f** Outer membrane fraction western blot with orange boxes highlighting the presence of OmpF and purple boxes for the presence of OmpC. An unspecific band of similar molecular weight is labelled with an asterisk. **g** Trace and retrace images of clear MG1655 surface features in five different areas on the same cell. The features are outlined with a white line. **h** Height and phase images of patches visualized in rough and clear *E. coli* OM. White-red, dashed lines indicate cross-sections for the line height profiles shown below. The red, dashed line refers to the patch region that is highlighted in pink in the line height profile. Patches are outlined with a white line in the phase and height images. Source data are provided as a Source Data file. Scale bars: 100 nm (**a**); 50 nm (**e**); 10 nm (**g**); 50 nm (**h**). Color (height/ phase) scales: 5 nm/ 2.2 deg (**a**); 5 nm (**e**); 2 nm (**g**); 5 nm/ 2.2 deg (**h**).

First, we compared rough and smooth *E. coli*, recording images using the same AFM tip in each biological repeat (Fig. 3a), to minimize tip-associated variability in the image quality. As expected, the absence or presence of the O-antigen did not significantly affect the detected number of pores, likely associated with outer membrane protein trimers, with 4 ~ 5 × 10^3^ per µm^2^ (Fig. 3b), consistent with previous studies of rough *E. coli*^7^. Moreover, both strains displayed smooth patches that appeared deficient in the pore network reminiscent of trimeric outer membrane proteins (Fig. 3a). These smooth patches were previously identified as LPS-enriched areas in the rough outer membrane^7^. The size (area) and shape (circularity) of these LPS-enriched patches were not found to have significant differences between rough and smooth *E. coli* (Fig. 3c, d).

By contrast, we observed major differences when imaging the clear *E. coli* strain, as per the schematic and data in Fig. 3e and Supplementary Fig. 3. Most remarkably, the AFM images of clear *E. coli* lacked the network of outer membrane protein (OmpF/OmpC) trimer features so characteristic of *E. coli*^7^. While biochemical analysis of outer membrane extracts confirmed reduced OmpF expression in clear MG1655, OmpC was present at similar surface levels as in rough MG1655 (Fig. 3f and Supplementary Fig. 4). Reduced OmpF expression may result either from the absence of oligosaccharide^30,44^ or from the stress favoring OmpC that reduces outer membrane permeability^45^.

Taking together, these observations indicate that while trimers are present in the clear *E. coli* outer membrane, they remain unresolved. This may be due to the local absence of polysaccharide directly above the trimers that provide AFM contrast through a local depression in rough *E. coli* (Fig. 3e) or due to higher membrane fluidity effectively blurring the AFM images. In AFM, resolution is affected by probe-sample interactions and the resulting contact area between the AFM probe and imaged features^46^. If these features rapidly diffuse during data acquisition, images may appear blurred. We note, however, that membrane fluidity is less plausible as an explanation for the lack of AFM contrast on clear *E. coli* since this contrast was not improved by chemical fixation (Supplementary Fig. 5).

Only rarely did we observe features in the clear *E. coli* membrane that appeared similar in size and appearance to the pores we associate with trimers of OmpF/OmpC (Fig. 3g and Supplementary Fig. 6), suggesting the formation of higher-order supramolecular assembly of porins such as previously observed on mitochondrial outer membranes^47^. While the interpretation of these features remains speculative, their similarity in trace and retrace images rules out common AFM artefacts.

Although the trimer networks appeared largely absent in AFM images of clear *E. coli*, the data did show feature-less domains similar to the LPS-enriched patches observed in rough and smooth *E. coli* (Fig. 3h). However, whereas these patches were higher than the protein trimer networks in the rough *E. coli* outer membrane, the patch height was below the surrounding surface area in the clear *E. coli* outer membrane. This is consistent with our explanation of the AFM trimer contrast above (illustrated in Fig. 3e), with the core polysaccharide, if present, extending well beyond the outer membrane protein surface. This also provides additional evidence for the biochemical identification of the featureless patches as LPS-enriched phases embedded in an outer membrane protein network.

### Reversible covalent immobilization facilitates AFM imaging across bacterial species

To test how the glutaraldehyde-based immobilization strategy translates to other bacterial species, we applied it to the Gram-negative *P. aeruginosa* PAO1, to a capsule-free mutant of the Gram-negative *A*. *baumannii* AB5075^*ptk*^, and to the Gram-positive *S. aureus* ATCC 29213, which are all ESKAPE species associated with high virulence and antimicrobial resistance^48^. In contrast to *E. coli*, *P. aeruginosa* PAO1 lacks unspecific large outer membrane porins and features diverse O-antigens of two different forms and various chain lengths^49,50^. *A. baumannii* commonly produces a protective capsule, but deletion of the *ptk* gene in *A. baumannii* strain AB5075 prevents the export of capsule polymers to the cell surface, resulting in an unencapsulated mutant strain and exposing the underlying outer membrane^51^. This membrane surface primarily features monomeric outer membrane porins and lipooligosaccharides mostly lacking O-antigen^52,53^.

**Fig. 4:**
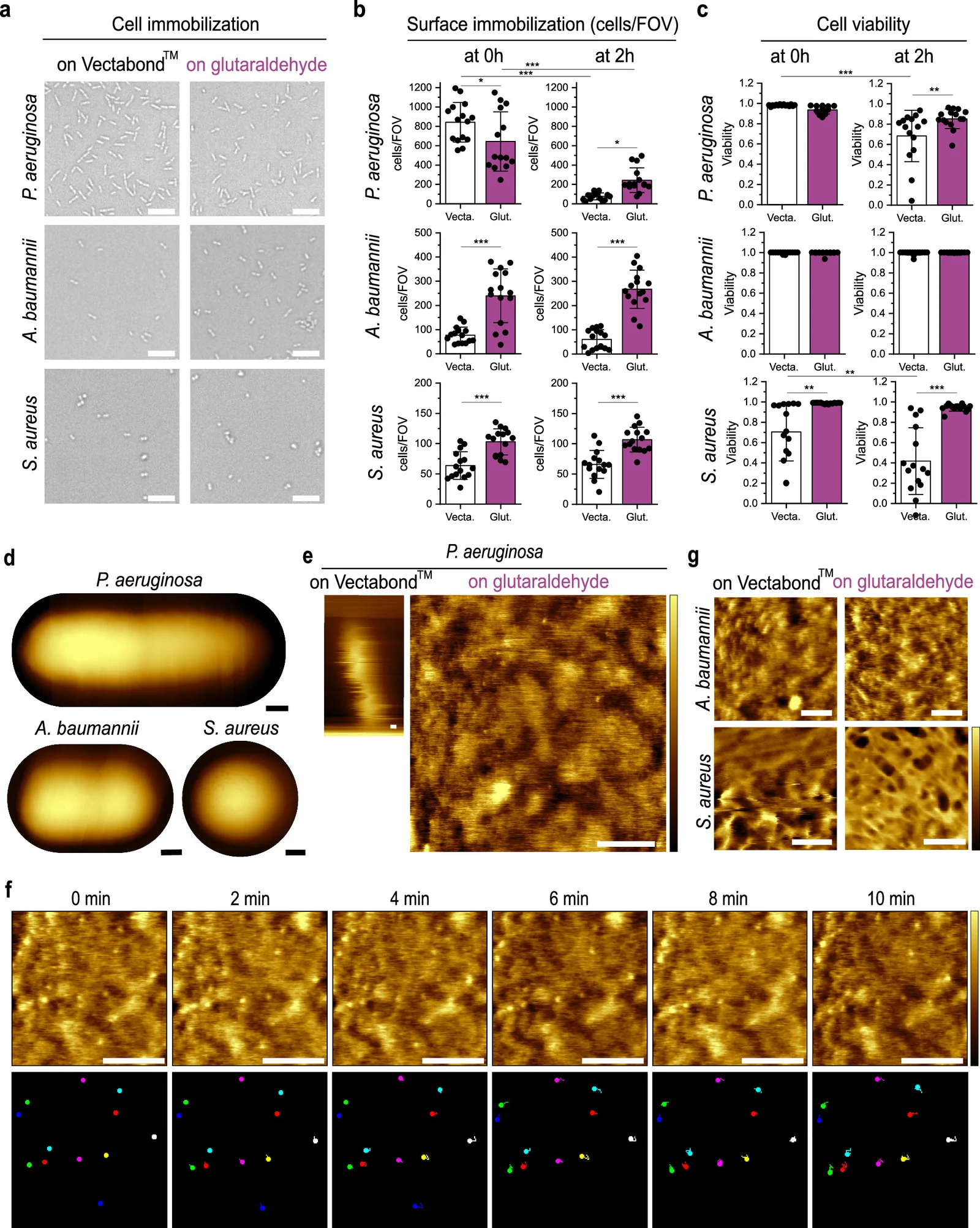
Covalent immobilization for live-AFM imaging across different pathogenic species. Immobilization efficiency (**a**, **b**), viability (**c**), and AFM images (**d**–**f**) of *P. aeruginosa* PAO1, capsule-less *A. baumannii* AB5075^ptk^, and *S. aureus* ATCC 29213 were studied on glass coverslips functionalized with Vectabond^TM^ or glutaraldehyde. **a** Bright-field images of immobilized cells on coated coverslips. Quantification of surface immobilization as cells/FOV, where the FOV measured 190 × 138 µm^2^ (**b**), and cell viability as the percentage of cells with maintained cell envelope integrity (**c**) of bacteria adhering to the functionalized coverslips immediately after immobilization (at 0 h) and 2 hours after immobilization and additional surface rinsing (at 2 h). Data points were determined from three biological repeats with five different acquisition areas (*n* = 15). Data are displayed as mean ± s.d. Statistical significance of differences was determined by two-way repeated-measures ANOVA, followed by Holm-Sidak multiple-comparison tests, with significant differences between the means of two groups highlighted with asterisk: *p* < 0.05 = *, *p* < 0.005 = **, *p* < 0.0005 = ***. **d** AFM height images of whole cells immobilized on glutaraldehyde-functionalized glass coverslips. **e** AFM height images of *P. aeruginosa* immobilized on Vectabond^TM^ and glutaraldehyde-treated slides. **f** AFM height image series of *P. aeruginosa* PAO1 nanoscale surface protrusions (top) and extracted tracks (bottom) imaged on the same cell over time (*n* = 1). **g** AFM height images of *A. baumannii* and *S. aureus* immobilized on Vectabond^TM^ and glutaraldehyde functionalized slides. Source data are provided as a Source Data file. Scale bars: 10 µm (**a**); 200 nm (**d**); 100 nm (**e**); 100 nm (**f**); 100 nm (**g**). Color (height) scales: 300 nm (**d**, *P. aeruginosa*); 300 nm (**d**, *A. baumannii*); 250 nm (**d**, *S. aureus*); 200 nm (**e**, on Vectabond^TM^); 20 nm (**e**, on glutaraldehyde); 4 nm (**f**); 6 nm (**g**, *A. baumannii*); 10 nm (**g**, *S. aureus*).

Glutaraldehyde-based immobilization outperformed the charge-based Vectabond^TM^ functionalization for all species by yielding higher or more durable cell attachment (Fig. 4a, b). Our results with PAO1 on Vectabond^TM^ are consistent with previous reports of poor or weak immobilization of *P. aeruginosa*^27^, with an order-of-magnitude drop in cell count after 2 h and an additional washing step, whereas on glutaraldehyde a more sizeable fraction (~1/3) remained. Additional optimization experiments showed that stable PAO1 attachment was maintained over 2 h on glutaraldehyde coating concentrations of 1-8%, whereas attachment decreased substantially at 0 and 0.5% (Supplementary Fig. 7). *A. baumannii* and *S. aureus* cells showed durable immobilization for both protocols, but 2 ~ 5 times higher cell numbers for glutaraldehyde than for Vectabond^TM^ (Fig. 4b). Cell viability was high in the assays with the Gram-negative pathogens *A. baumannii* and *P. aeruginosa*, with similar or slightly better viability on glutaraldehyde than for Vectabond^TM^ (Fig. 4c). In contrast, for the Gram-positive *S. aureus*, only glutaraldehyde yielded robust viability, close to 100%.

Surface immobilization on glutaraldehyde-functionalized slides facilitated high-resolution AFM images of living *P. aeruginosa* (Fig. 4d, e). *P. aeruginosa* appeared stable under AFM imaging and showed height variations in the form of surface protrusions that range in size between <5 and 20 nm (Fig. 4e–f and Supplementary Fig. 8, Supplementary Fig. 9). By contrast, the problems of poor or transient immobilization were illustrated by AFM imaging on Vectabond^TM^-adhered *P. aeruginosa*, with the cells appearing fuzzy and streaky even at low magnification, or even entirely detaching (Fig. 4e, inset), precluding any high-resolution AFM. The *P. aeruginosa* outer membrane surface is clearly distinct from *E. coli*: The AFM images do not show the networks of pores associated with trimeric outer membrane protein assemblies in *E. coli*. This is not unexpected, given the low expression of trimeric porins in *P. aeruginosa*^49,54^. In addition, the complex LPS of *P. aeruginosa*^50^ may sterically prevent visualization of outer membrane components. Surface images of *P. aeruginosa* did reveal small surface protrusions of <5 nm height whose nature and origin remain unclear (Fig. 4e–f and Supplementary Fig. 9).

Imaging these protrusions over time allowed us to estimate the diffusion coefficient of (presumably) proteinaceous contents in the *P. aeruginosa* outer membrane. It was found to be at the border of the localization noise floor, and estimated to lie between ~0.36 × 10^−7^ µm^2^ ⋅ s^−1^ and ~0.44 × 10^−7^ µm^2^ ⋅ s^−1^ (Supplementary Fig. 10), similar to previous estimates for the diffusion coefficient for porin trimers in the *E. coli* outer membrane (*D* ~ 2 × 10^−7^ µm^2^ ⋅ s^−1^)^7^. This indicates that the outer membrane of *P. aeruginosa* has a similarly immobile (or highly viscous) nature as previously found for *E. coli*.

For *A. baumannii* and *S. aureus*, both immobilization strategies facilitated AFM imaging of whole cells, shown on glutaraldehyde in Fig. 4d, with accordingly higher-resolution images in Fig. 4g. Further images are provided in Supplementary Fig. 11 and Supplementary Fig. 12. In practice, AFM imaging of *A. baumannii* appeared similar for both functionalizations, whereas for *S. aureus*, AFM imaging appeared to be more robust for glutaraldehyde-immobilized cells. As for *P. aeruginosa*, the outer membrane of *A. baumannii* appears qualitatively different from that of *E. coli*, lacking characteristic trimeric protein features. Images of living *S. aureus* (without outer membrane) revealed a mesh-like peptidoglycan cell wall arrangement with regions of concentric rings, as found previously on whole cells and purified cell wall peptidoglycan (sacculi)^11^ .

### Nanometer-resolution mapping of the poles of rod-shaped bacteria

**Fig. 5:**
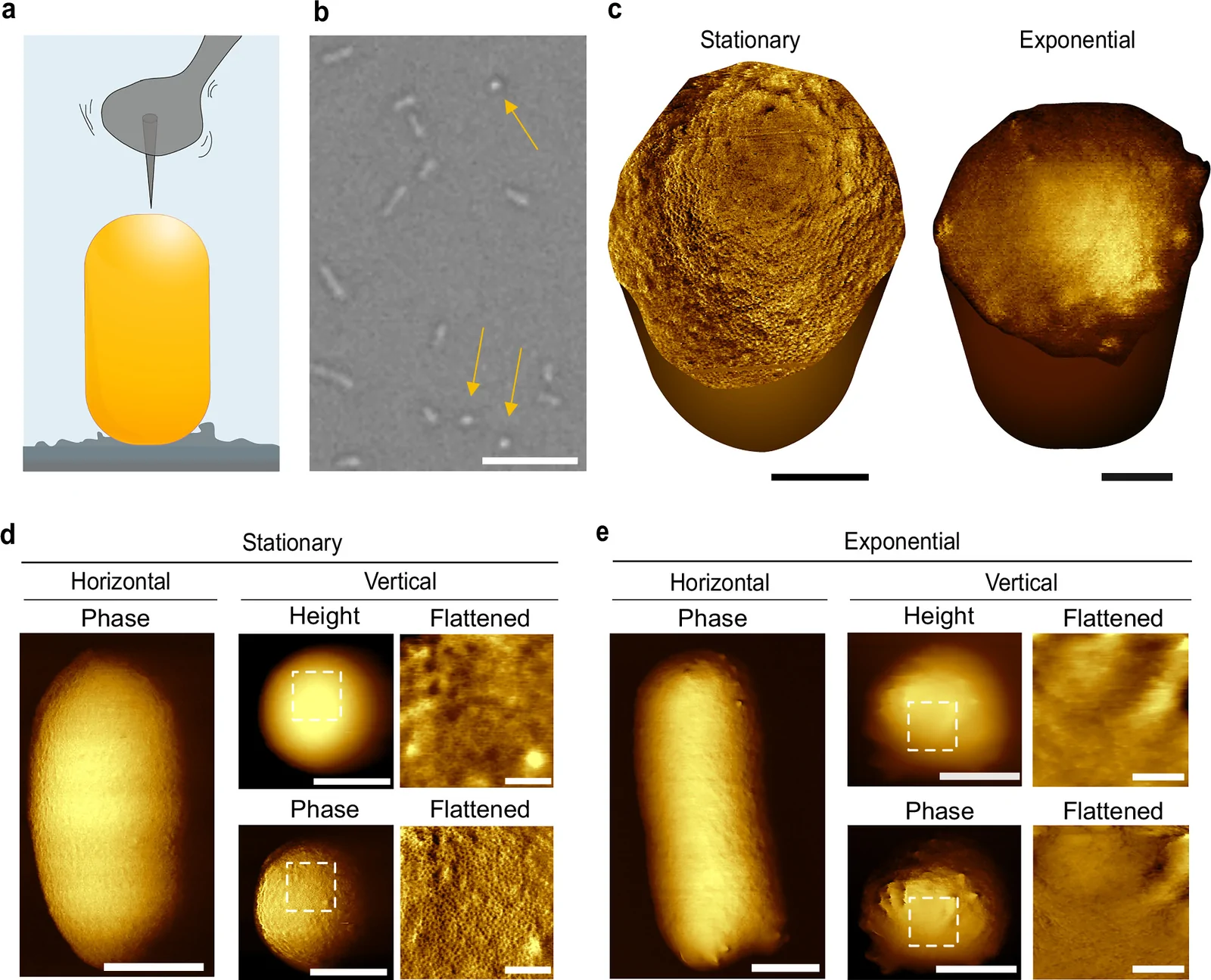
Prolonged covalent attachment facilitates high-resolution imaging of bacterial poles. **a** Cartoon of AFM imaging on bacterial poles. **b** Bright field image of immobilized cells incubated for 50 min on glutaraldehyde-functionalized coverslips. Yellow arrows indicate apparently round, hence presumably vertically oriented bacteria, presenting their poles to optical microscopy and to the AFM tip. **c** AFM phase images of the poles of stationary and exponentially growing *E. coli* MG1655 cells. AFM images of stationary (**d**) and exponentially grown (**e**) *E. coli* cells in horizontal and vertical orientations, with raw phase and height data, as well as flattened magnifications for the dashed white outlines on the cell poles. Scale bars: 10 µm (**b**); 200 nm (**c**); 500 nm (**d**, **e**, whole-cell views); 100 nm (**d**, **e**, flattened). Color (height/ phase) scales: 3.3 deg (c, stationary); 6.5 deg (**c**, exponential); 10 deg (**d**, **e**, horizontal); 250 nm/ 9 deg (**d**, **e**, stationary, vertical, whole cell); 400 nm/ 10 deg (**d**, **e**, exponential, vertical, whole cell); 10 nm/ 2.8 deg (**d**, **e**, stationary, flattened); 20 nm/ 2 deg (**d**, **e**, exponential, flattened).

As also exemplified by *E. coli, P. aeruginosa* and *A. baumannii* above, many bacteria are rod-shaped and are commonly immobilized with their long axis parallel to the AFM substrate. Since the AFM tip is oriented approximately perpendicular to the AFM substrate, the poles of immobilized bacteria are thereby inaccessible to the AFM tip. The same limitation also applies to most optical microscopy experiments^55^. To enable AFM access to bacterial poles (Fig. 5a), we explored different incubation times of bacteria on glutaraldehyde-functionalized coverslips. Longer incubation (50 min) resulted in attachment of *E. coli* MG1655 in orientations that appeared to be both parallel and perpendicular to the coverslip substrate (Fig. 5b). Presumably, the prolonged incubation times increase covalent bond formation between bacteria and the glass surface, strengthening attachment. Local variations in aldehyde density may further promote vertical immobilization.

Although we also observed cases where the AFM tip dislodged such vertically oriented cells and thereby revealed their rod-shape (Supplementary Movie 1), these cells were readily imaged by AFM at a similar resolution as horizontally oriented cells (Fig. 5c). This is of particular interest since bacterial poles represent what were previously division planes of mother cells, thereby likely containing information on processes and structures critical to cell division (cf. the different arrangements of the peptidoglycan cell wall as previously observed for the spherical, Gram-positive *S. aureus*)^11^. As a first exploration of this potential, we compared *E. coli* cells that were in different growth phases when immobilized on the cover slips.

In the stationary phase, when cells had depleted available nutrients and on average growth had stalled, cells presented smooth, round poles with dense networks of trimeric outer membrane protein features (Fig. 5c, d), as observed elsewhere on the cell surface (Figs. 2f, 3a, e, 5d, c)^7^. By contrast, when cells were harvested and immobilized from the exponential growth phase, poles commonly showed morphological differences compared to the smooth stationary hemispherical pole shape, and the porous features reminiscent of outer membrane protein trimers remained unresolved (Fig. 5c, e and Supplementary Fig. 13).

## Discussion

We have established a method that facilitates label-free, live-cell, nanometer-scale imaging of cell envelopes for a wide range of bacterial species. To do so, we adopted and adjusted protocols that had previously been used for optical imaging of rough *E. coli* on glass^35^ and for AFM imaging of rough *E. coli* and of *Magnetospirillum magneticum* and *Rhodobacter sphaeroides* on mica substrates^12,13^.

For most protocols used previously, there are large variations in bacterial immobilization and associated viability as a function of species^35^, also exemplified by recently reported difficulties with immobilizing living *P. aeruginosa*^27^ and by our own attempts of immobilizing/imaging smooth *E. coli* (see Fig. 2)^7^. Glutaraldehyde-functionalized surfaces can overcome this problem by reversible covalent binding between the AFM substrate and bacterial surface amines.

Remarkably, and certainly compared with charge-based immobilization (e.g., Vectabond^TM^), glutaraldehyde functionalization provided a more robust method to immobilize bacteria for high-resolution AFM imaging across strains and species, whilst the bacterial viability remains largely unaffected, to the point that immobilized rough *E. coli* can grow and divide on the substrate (Figs. 2 and 4).

Using this functionalization to compare clear, rough and, previously inaccessible, smooth *E. coli*, we find no significant differences in the AFM images between rough and smooth strains, both showing the characteristic networks of trimeric porins (appearing via local depressions in the bacterial surface) interspersed by protein-free domains (Figs. 2, and 3)^7^. However, for the clear *E. coli* (Fig. 3), with complete absence of LPS polysaccharide, the contrast is much degraded, strongly suggesting that AFM resolves the porin trimers indirectly through a depression by the local absence of polysaccharide chains directly above trimers. Alternatively, the membrane fluidity of clear *E. coli* may be affected by the absence of polysaccharide and the additional use of lipid IV(A) with 4 acyl chains, which, with increasing membrane fluidity, could affect AFM resolution. In addition, whereas the smooth and rough strains show protein-free nanoscale domains that appear to protrude from the surface^7^, the clear *E. coli* shows similar featureless, smooth domains with a surface beneath the surrounding, more corrugated and thereby presumed protein-rich areas (Fig. 3). This reinforces the previous interpretation^7^ of these domains as LPS-enriched islands.

Focusing next on species that were previously not or less amenable to live-cell AFM imaging (Fig. 4), we observe that the outer membranes of *P. aeruginosa* and *A. baumannii* lack the pronounced networks of pores, associated with trimeric porins^7^, seen in AFM images of *E. coli*. This is expected because of the absence of large general porins and low copy numbers of protein trimers in *P. aeruginosa* and *A. baumannii* ^49,52,54^. Generally, however, these outer membranes do show nanometer-scale undulations or possibly domains, the origin of which remains open for interpretation. Nonetheless, by their static appearance within the AFM acquisition time of approximately 3 min, these features indicate that *P. aeruginosa* and *A. baumannii* outer membranes have a similar lack of fluidity as the *E. coli* outer membrane^7,8^. Our method also benefitted high-resolution AFM imaging of Gram-positive bacteria, as shown by the example of *S. aureus*, which in our live-cell data showed a structure highly similar to peptidoglycan architectures previously reported^11^ .

While 2% glutaraldehyde coatings provided robust attachment for all bacterial species tested here, coating conditions may benefit from further optimization for specific organisms and experimental requirements, as attachment efficiency can vary with cell type and assay conditions. Generally, different immobilization approaches may preferentially facilitate the attachment of certain bacteria within a population and, consequently, there is a risk that they may not capture the full heterogeneity of the bacterial population.

Finally, with minor adaptation of incubation conditions, the glutaraldehyde-based immobilization is found to be sufficiently strong to immobilize rod-shaped bacteria in a vertical orientation, making their poles accessible for high-resolution AFM (and optical) imaging (Fig. 5). This is relevant because several antimicrobials have been reported to preferentially interact with bacterial pole regions, such as the membrane attack complex^56^, cationic-hydrophobic nanoparticles^57^, and certain bacteriophages^58^. Moreover, this provides access to surfaces that directly originate from the division plane of mother cells. As shown here for *E. coli*, the poles of stationary-phase bacteria had similar outer membrane protein networks as elsewhere on the surface. In contrast, for exponential-phase bacteria (more likely to have recently undergone cell division^59^), these networks are unresolved or absent, and additional morphological defects are more common. These differences may be indicative of cell envelope restructuring and biosynthesis during and/or immediately after cell division and further emphasize the potential of our immobilization methods to open new research avenues to investigate fundamental biological processes.

Taken together, our method widens the scope of nanometer-resolution live-cell imaging of bacterial cell envelope structures, from rough *E. coli*^7–9,28^ and the Gram-positive *S. aureus*^10,11,17^ to other ESKAPE bacteria, including (for Gram-negative bacteria) different levels of LPS complexity (e.g., ±- O-antigen). This will benefit fundamental studies into cell envelope architecture and restructuring, as well as analyses of cell envelope disruption or degradation by (existing or novel) antibiotics. Our approach may also be of interest to study processes occurring at longer timescales, such as successive cell divisions and biofilm formation, which remain to be tested.

## Methods

### Strains and culture conditions

Overnight cultures of *E. coli* MG1655 (“rough”), *E. coli* MG1655 *wbbL*^*+*^ (“smooth”), *P. aeruginosa* PAO1 ATCC-BAA-47 HER-1018, *A. baumannii* AB5075^ptk^, and *S. aureus* ATCC-29213 were grown from single colonies in 2–3 mL of growth media at 37 °C and 220 rpm. *E. coli* MG1655 and *E. coli* MG1655 *wbbL*^*+*^ were grown in Mueller-Hinton broth (MHB), *P. aeruginosa* PAO1, ATCC-BAA-47, and *A. baumannii* AB5075^ptk^ were grown in Luria-Bertani (LB) broth; and *S. aureus* ATCC-29213 was grown in Tryptic Soy Broth (TSB). ClearColi™ KPM 318 (K12) (*msbA52 ∆kdsD ∆gutQ ∆lpxL ∆lpxM ∆pagP ∆lpxP ∆eptA*) (“clear”) was directly grown from a glycerol stock, and *Escherichia coli* MG1655, used for comparison, was grown from a single colony, both in 3 mL of LB broth at 37 °C and 240 rpm. For all cell lines, the overnight cultures (stationary cells) were diluted 1:100 in 3-5 mL fresh growth media and regrown until reaching mid-logarithmic phase at OD_600_ of 0.5-2.7.

### Strain construction

RKCK29 (MG1655 Δ*ompF*) was constructed using a modified High-efficiency Scarless Lamda Red Recombination method^60^. In short, spectinomycin resistance cassette flanked by I-SceI restriction site and 400 bp of down- and upstream of *ompF* was electroporated into *E. coli* MG1655 expressing λ-Red recombinase from the pREDTKI plasmid (a gift from Sheng Yang (Addgene plasmid # 51628; http://n2t.net/addgene:51628; RRID: Addgene_51628)). The spectinomycin resistance cassette was excised by digestion with IPTG-inducible I-SceI followed by a λ-Red recombination event. Sequencing results confirmed the intended deletion. The process was repeated for RKCK30 (MG1655 Δ*ompF* Δ*ompC*), using RKCK29 as a parent strain.

### Coverslip cleaning

Coverslips (Ø 13 cm, #1.5, Epredia) used as substrate for bacterial immobilization were cleaned as reported by Benn et al. (2019)^28^. The coverslips were rinsed with Milli-Q water and sonicated in 1% SDS at 37 kHz and 100% power in a Fisherbrand™ bath sonicator (Fisher Scientific) for 10 min. This was followed by rinsing with Milli-Q water, ethanol, and drying under a continuous nitrogen stream. The coverslips were transferred into a ceramic coverslip holder and air-plasma cleaned at ~0.3 mbar and 70% power for 2 min (Zepto, Diener Electronic). This procedure was repeated one more time. Cleaned coverslips were stored at room temperature for up to one week.

### Vectabond^TM^ coating

Vectabond^TM^ coating was performed as described by Benn et al. (2019)^28^. Cleaned coverslips were transferred into a ceramic coverslip holder and activated with air plasma at ~0.3 mbar and 70% power for 2 min. The coverslips were immediately transferred into a solution of 1:50 v:v of Vectabond^TM^ (Vector Laboratories, USA) and acetone and incubated for 5 min. This was followed by rinsing with milli-Q water and drying under a continuous nitrogen stream. Prior to bacterial immobilization, the coverslips were glued onto the center of a glass microscope slide (Brand) using Reprorubber® Thin Pour (Flexbar), thereby also providing a rim to confine a liquid bubble of up to approximately 200 μL.

### Glutaraldehyde coating

Glutaraldehyde functionalization of glass coverslips was inspired by Syga et al. (2018)^35^, with minor adjustments to yield a protocol as follows. First, cleaned coverslips were transferred into a ceramic coverslip holder and activated with air plasma at ~0.3 mbar and 70% power for 2 min. Directly after plasma cleaning, the coverslips were transferred into a coverslip holder and immersed in 2% APTES (v/v in acetone, ThermoFisher) for 2 min. The coverslips were next washed 3 times with acetone and dried under a continuous nitrogen stream. Previous to bacterial immobilization, the coverslips were glued into the center of a glass slide using Reprorubber® Thin Pour. Upon glue solidification, 2% glutaraldehyde solution (100 μL), prepared in Milli-Q water (4 °C, pH 5.5) and stored at −20 °C until use, was added to the coverslips and incubated for 30 min at 4 °C. The low temperature and slightly acidic environment minimize polymerization of glutaraldehyde, which would otherwise reduce the immobilization efficiency. To ensure no free glutaraldehyde remained in the solution for bacterial incubation, the glutaraldehyde solution was removed by rinsing 5-10 times with 1 mL Milli-Q water, where caution was taken not to let the glass surface dry out before adding the suspension with the bacteria to be immobilized.

### Lectin functionalization

Glutaraldehyde-functionalized coverslips were prepared as described above. After coverslip rinsing with Milli-Q water, 100 μL of 1 mg/mL Concanavalin A (ConA, Cambridge Bioscience), prepared in HEPES (20 MM, pH 7.4) buffer, was incubated for 10 min at room temperature. The surface was rinsed 3 times with 1 mL Milli-Q water before being used as the substrate for bacterial immobilization.

### Bacterial immobilization

Bacteria were prepared in 500 μL of 0.5 OD₆₀₀ culture and washed twice by centrifugation (Supplementary Tab.1) and resuspension in 500 μL minimal medium without glucose (MM; 1× M9 salts (ThermoFisher Scientific), 2 mM MgSO₄, 0.1 mM CaCl₂). The final wash was performed with HEPES buffer (20 mM, pH 7.4) before resuspending the cells in 500 μL of fresh HEPES for immobilization on Vectabond^TM^ or glutaraldehyde-functionalized slides and HEPES+ (10 mM HEPES, 150 mM NaCl, pH 7.4) for lectin-coated slides. *A. baumannii* cells were centrifuged once (Supplementary Tab.1), the supernatant removed, and the pellet directly resuspended in HEPES for immobilization. 100 μL of the cell suspension was incubated on prepared coverslips for 5 min on Vectabond^TM^-coated surfaces, 10 min on lectin-coated slides, and 20 minutes on glutaraldehyde-coated slides. For AFM imaging of cellular poles, bacteria were immobilized for 50 min on glutaraldehyde-coated slides. The slides were next washed three times with 1 mL MM, before being replenished with 200 μL MM for optical microscopy and/or AFM experiments. 2 μL of SYTOX^TM^ (final concentration 5 μM) was added for assessment of bacterial cell viability, or 1 μL of propidium iodide (PI; final concentration 15 μM) for *S. aureus*.

### Bacterial fixation to study the effect of membrane fluidity

Clear and rough *E. coli* were fixed according to a protocol by Klein et al. (2020)^61^. In short, 200 µL of overnight cultures were collected through centrifugation at 2150 × *g* for 2 min and chemically fixed by resuspension in 4% paraformaldehyde, 0.1% glutaraldehyde, and 0.1 M PHEM buffer pH 7.4 (60 mM PIPES, 25 mM HEPES, 10 mM EGTA, 2 mM MgCl_2_) for 30 min at room temperature. Bacteria were washed 2x in PHEM buffer and immobilized on 2% glutaraldehyde-coated slides for 30 min.

### Optical microscopy to determine cell attachment and cell viability

Bacterial samples were prepared as described above and imaged with an Olympus IX 73 inverted optical microscope equipped with an Andor Zyla 5.5 USB3 fluorescence camera, and a white light pE-400 illumination system or an Olympus U-LH100HG fluorescence light source. For each sample, bright field and fluorescence images were acquired in five different sample positions. Fluorescence images were recorded with 10 ms excitation time and 20% laser power. The samples were stored in a box covered with aluminum foil for 2 h, then washed three times with MM before re-measurements of cell attachment and bacterial viability were performed with fresh SYTOX^TM^ or PI. Surface immobilization was quantified as the number of cells counted per FOV using the ImageJ tool “Find Maxima,” setting the prominence between 7–10 (237 × 178 µm^2^ for quantifications in Fig. 2 and 190 × 138 µm^2^ for quantifications in Fig. 4), and cell viability was quantified as 1 minus the proportion of SYTOX^TM^- or PI‑positive cells, setting the prominence to 10.

### AFM imaging and data processing

AFM images displayed in Figs. 2, 3e, f were acquired at room temperature using a NanoRacer AFM head mounted on an inverted optical microscope with an additional sample scanner to facilitate larger-area scanning (Bruker, Berlin) using AC mode with FastScanD cantilevers. The other AFM images were acquired on a Nanowizard III AFM with UltraSpeed head (Bruker AXS) using AC mode with FastScanD cantilevers. The cantilever excitation frequency was chosen between 85 and 140 kHz, close to the cantilever resonance, with a setpoint between 50 and 80% relative to a free amplitude of 20 nm as measured far away from the surface. Images were acquired at 512 × 512, 512 × 256, or 256 × 256 pixel ratio, at a line rate of 1–5 Hz, resulting in a maximum acquisition time of 8 min and 32 s per image. Images for display were processed using Gwyddion 2.68^62^. Unless otherwise stated as “raw” image, height and phase images were first mean-plane subtracted, then base-flattened, followed by second-order polynomial flattening, and a Gaussian filter of 0.5–1 pixel width was applied.

To quantify outer membrane protein timers for Fig. 3b, 300 × 300-pixel (293 × 293 nm) data, cropped from phase images, were analyzed using Fiji^63^, as follows. Raw data were processed as previously described, applying a 1-pixel Gaussian filter. In Fiji, the images were auto-thresholded, and pores were detected using the built-in “Find Maxima” function, which identifies local maxima using a neighborhood maximum filter followed by flood-fill pruning based on a prominence threshold (see https://github.com/dwaithe/MaximaFinder). The “Find Maxima” operation on a “light background” finds local minima (the observed pores in the outer membrane of phase images), setting the prominence to 30 and using the “excluding edges” option. The prominence level of 30 was found, by visual inspection, to identify the majority of pores. Example repeats of the acquired phase images and the extracted pores are shown in Supplementary Fig. 14. Line profiles of height images in Fig. 3h, Supplementary Figs. 6, 9b were extracted with Gwyddion 2.68.

A custom-written Python script was used to quantify the patch locations and shape characteristics. This took as input the coordinates of porin trimers and calculated their Delauney triangulation using the Shapely package. A maximum edge separation of 27.5 nm was used to define the patch areas—it was empirically verified that this successfully captured the porin-free regions on the OM. The patch area and circularity were then computed for 3 scans from 3 biological repeats each for the rough and smooth *E. coli* strains. See https://github.com/hoogenboom-lab/bacteria-imaging for the processing script. An example pipeline output image is displayed in Supplementary Fig. 15. Patches that extended beyond the image boundaries and patches of surface areas less than 1500 nm^2^ were not included in the analysis. Only patches that, upon visual inspection, were confirmed to lack porin-like features were included for quantification. Data points were acquired from three biological repeats, imaging three cells for each preparation.

The diffusion coefficient (D) for the outer membrane of *P. aeruginosa* PAO1 was determined from a time-lapse image series recorded at 114 s per frame for 10 min. PAO1 cells were grown in Casamino acid medium (0.5% w/v Casamino acid, 5.2 mM K_2_HPO_4_, pH 7), supplemented with 1 mM MgSO_4_ and 0.1 mM CaCl_2_. For feature tracking, images were processed as previously described, cropped to 250 × 250 nm (160 × 160 pixels), and subjected to additional second-order flattening. Features were identified and tracked manually from frame to frame using the Fiji Manual Tracking plugin ^63^. For each pair of feature positions at different lag times (*τ*), the mean square displacement (MSD, μm^2^) was calculated and plotted against τ (s). The MSD versus τ data were fitted using both a linear fit constrained through zero and an unconstrained fit. The extracted slopes (m) were used to determine the diffusion coefficient according to *D* = m/4, assuming two-dimensional normal diffusion.

### Western blot

Overnight 5 mL LB cultures were grown from −80 °C glycerol stocks. An aliquot of 500 μL was used to start 50 mL LB, and cultures were grown at 37 °C with 190 rpm shaking to OD_600_ 0.5. Cells were harvested (10 min, 10,000 × *g*, 4 °C), and pellets were frozen at –80 °C. The outer membranes were extracted according to Housden et al. 2005^64^, with modifications. In short, cell pellets were resuspended in 35 mL of 10 mM Tris-HCl, pH 8.0, 0.25 % (w/v) lithium diiodosalicylic acid buffer with 1 mM phenylmethylsulfonyl fluoride to inhibit protease activity. Samples were sonicated 3 s on/ 7 s off in ice/water for a total process time of 2 min, using the stud probe on the Sonicator 4000 at amplitude 7. Samples were mixed thoroughly by inversion and sonicated for a further 2 min. The insoluble cell debris was removed through centrifugation (20 min, 10,000 × *g*, 4 °C). Pellets were discarded, and 25 mL of supernatant was centrifuged (200,000 × *g*, 4 °C, 45 min). The total membrane pellet was resuspended in 3 mL of 10 mM Tris-HCl, pH 8.0, 0.25% (w/v) LIS, 2% (v/v) Triton X-100 buffer. Samples were homogenized using a manual homogenizer. Membrane fractions were pelleted (200,000 × *g*, 4 °C, 45 min) to remove the inner membrane fraction. The membrane pellet was resuspended in 3 mL of 10 mM Tris-HCl, pH 8.0, 0.25% (w/v) LIS, 2% (v/v) Triton X-100 buffer, homogenized, and centrifuged again. The membrane pellet was resuspended in 3 mL of 10 mM Tris-HCl, homogenized, and centrifuged to remove the detergents. Final outer membrane sample was resuspended in 0.5 mL 10 mM Tris-HCl, pH 8.0, 2% (w/v) n-octyl-β-D-glucopyranoside (β-OG), 5 mM EDTA and homogenized. Half of the samples were boiled. All samples were kept frozen at −20 °C. 5 μL of each sample was run on a 10% SDS-PAGE gel and blotted onto a PVDF membrane. Membranes were blocked for 1 h in 8% milk in TBST, washed with TBST, and incubated with Anti-OmpF (Biorbyt, #orb752372, LOT-W8537, cross-reactive with OmpC) antibodies 1:1000 in 4% milk in TBST at room temperature overnight. Membranes were washed in TBST three times (5, 10, 5 min) and incubated with anti-rabbit antibodies in 4% milk in TBST at RT for 1 h. After three washes in TBST (5, 10, 5 min), signal was detected using ECL Select reagent. For a full scan blot, see the source data or Supplementary Fig. 4.

### Statistics and reproducibility

Experiments were performed on three biological replicates, unless otherwise stated. The resulting data are presented as the arithmetic mean with standard deviation of the mean. For single comparisons, a Mann-Whitney Test was used, and for multiple comparisons, two-way repeated-measures ANOVA, followed by Holm-Sidak multiple-comparison tests, was used. Details are provided in the figure legends. Asterisks on graphs indicate significant differences between data, and the precise *P* values for all comparisons are provided in the source data. Statistical analyses were performed using Origin 2024b or GraphPad Prism 10.

### Data visualization

Graphs were plotted using Origin 2024b and GraphPad Prism 10. Figure illustrations were created, and figures were formatted using Affinity Designer 1.10.6.

### Reporting summary

Further information on research design is available in the Nature Portfolio Reporting Summary linked to this article.

## Supplementary information

Supplementary Information

Description of Additional Supplementary Files

Reporting Summary

Supplementary Movie 1

Transparent Peer Review file

## Source data

Source Data

## Data Availability

The data generated in this study have been deposited in the UCL Research Data Repository database under accession code 33416257. Source data are provided with this paper.

## Code availability

The source code *“porin_patch_analysis.py”* for the analysis of the patch locations and shape characteristics can be found in the GitHub repository: https://github.com/hoogenboom-lab/bacteria-imaging.
