# Supplementary material for: Nanometer-resolution imaging of living bacteria across species and cellular orientations
Source: Nat Commun. 2026 Nov 9;17:11329. doi: 10.1038/s41467-026-78975-y (PMC13748617; doi:10.1038/s41467-026-78975-y)
Supplement: Supplementary file 1 [file 41467_2026_78975_MOESM1_ESM.pdf]

Supplementary Information

Nanometer-resolution imaging of living bacteria across species and cellular orientations

Anna Scheeder[1,*], Joanna Szczepaniak[2], Yulianna Koziy[1], Naomi Mara Claro De Oliveira[1,3], William Trewby[1], Renata Kaminska[2], Jeremy Brown[3], Colin Kleanthous[2], Bart W. Hoogenboom[1,4,*]

[1] London Centre for Nanotechnology, University College London, London WC1H 0AH, United Kingdom

[2] Department of Biochemistry, South Parks Road, University of Oxford, Oxford OX1 3QU, United Kingdom

[3] UCL Respiratory, Division of Medicine, University College London, London, WC1E 6JF, United Kingdom

[4] Department of Physics & Astronomy, University College London, London WC1E 6BT, United Kingdom

* Email: a.scheeder@ucl.ac.uk; b.hoogenboom@ucl.ac.uk

Supplementary Table 1. Centrifugation parameters for bacterial preparation.

**Strain**	**Centrifugation speeds (x *g*)**	**Centrifugation duration (min)**
E. coli	2,150	2
P. aeruginosa	2,150	2
A. baumannii	2,150	2
S. aureus	2,150	1.5

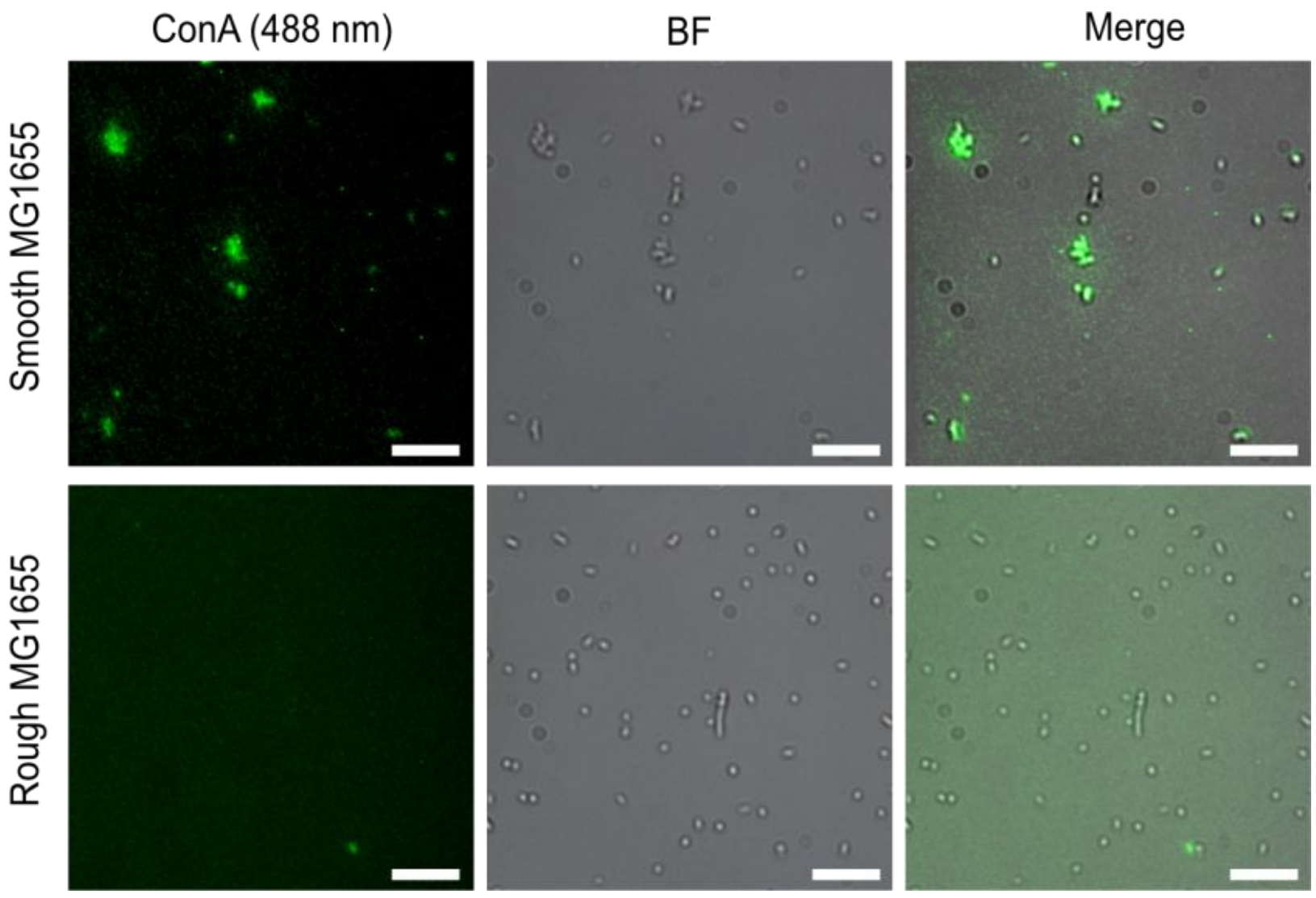

Supplementary Fig. 1. Bright field and fluorescence microscopy images of Concanavalin A targeted bacteria. Smooth and rough MG1655 were incubated for 15 minutes with Alexa Fluor 488 labeled Concanavalin A (ConA) prepared to a concentration of 1 mg/mL in HEPES buffer. ConA was specifically labeling smooth MG1655 cells while no significant association with rough MG1655 was observed. Scale bar: 10 µm.

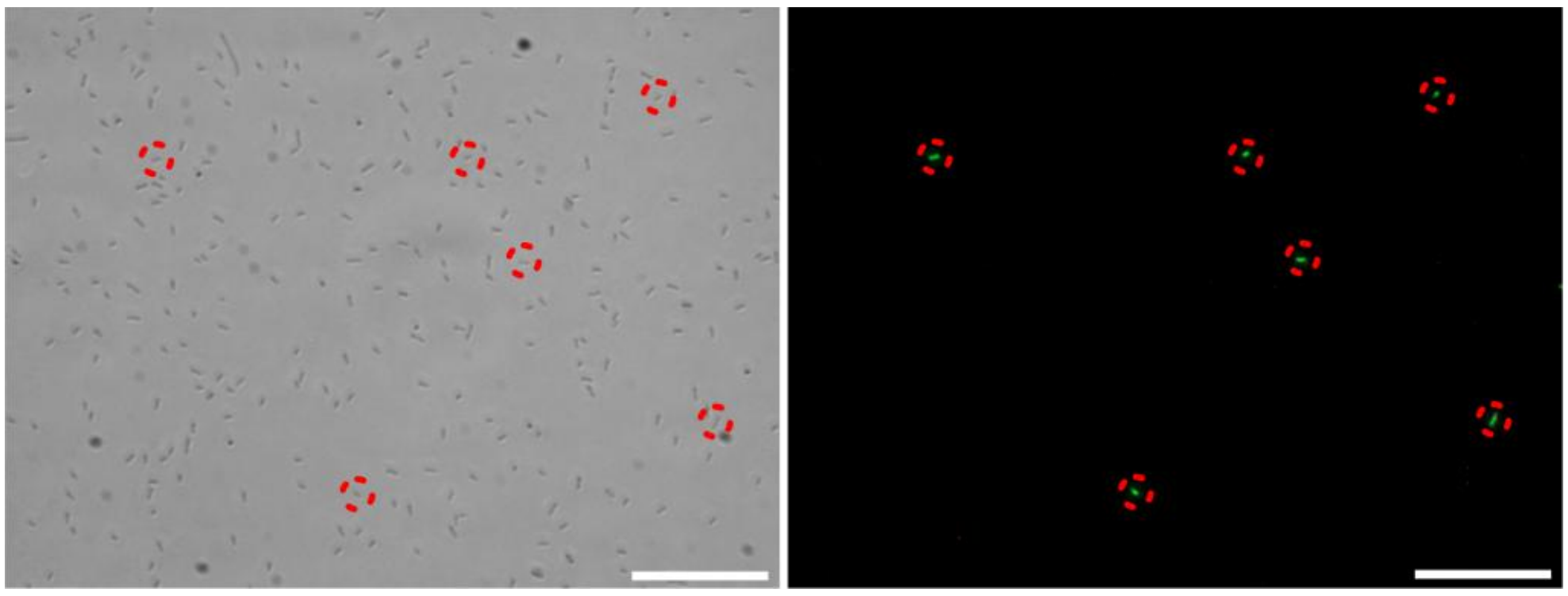

Supplementary Fig. 2. Bright-field and corresponding SYTOX^{TM}-positivity fluorescence images of rough *E. coli* immobilized on Vectabond^{TM}. SYTOX^{TM}-positive cells (right) are highlighted with red circles. Scale bar: 25 μm.

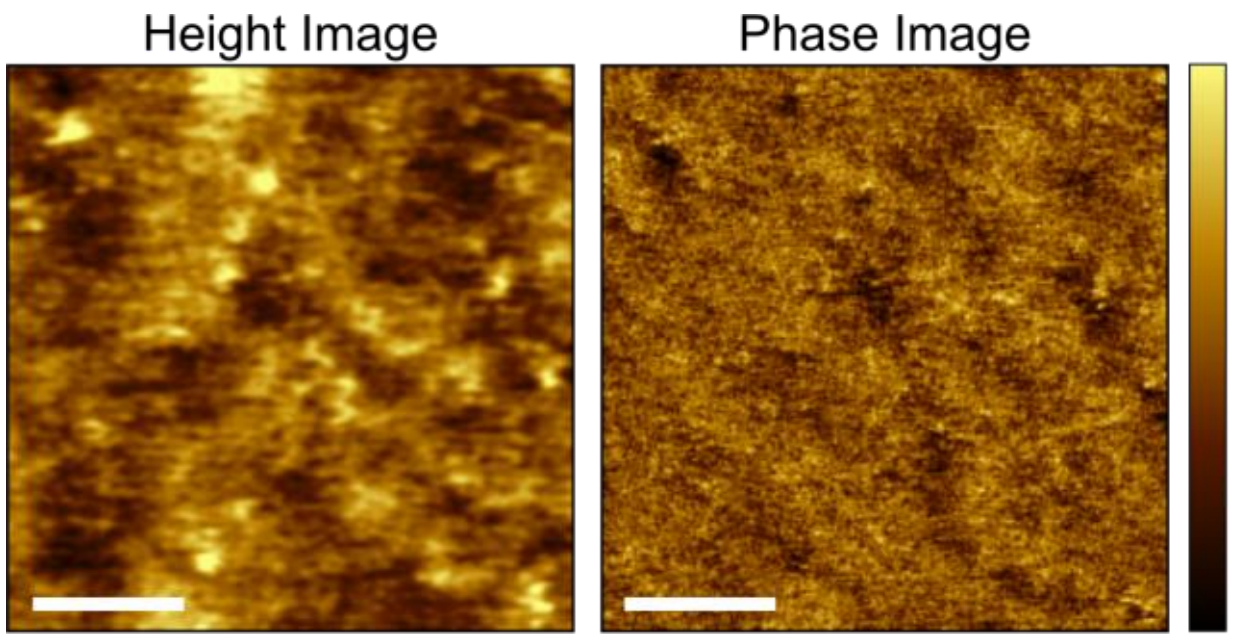

Supplementary Fig. 3. AFM height and corresponding phase images of clear MG1655.
Scale bars: 50 nm. Color (height/ phase) scales: 5 nm/ 1 deg.

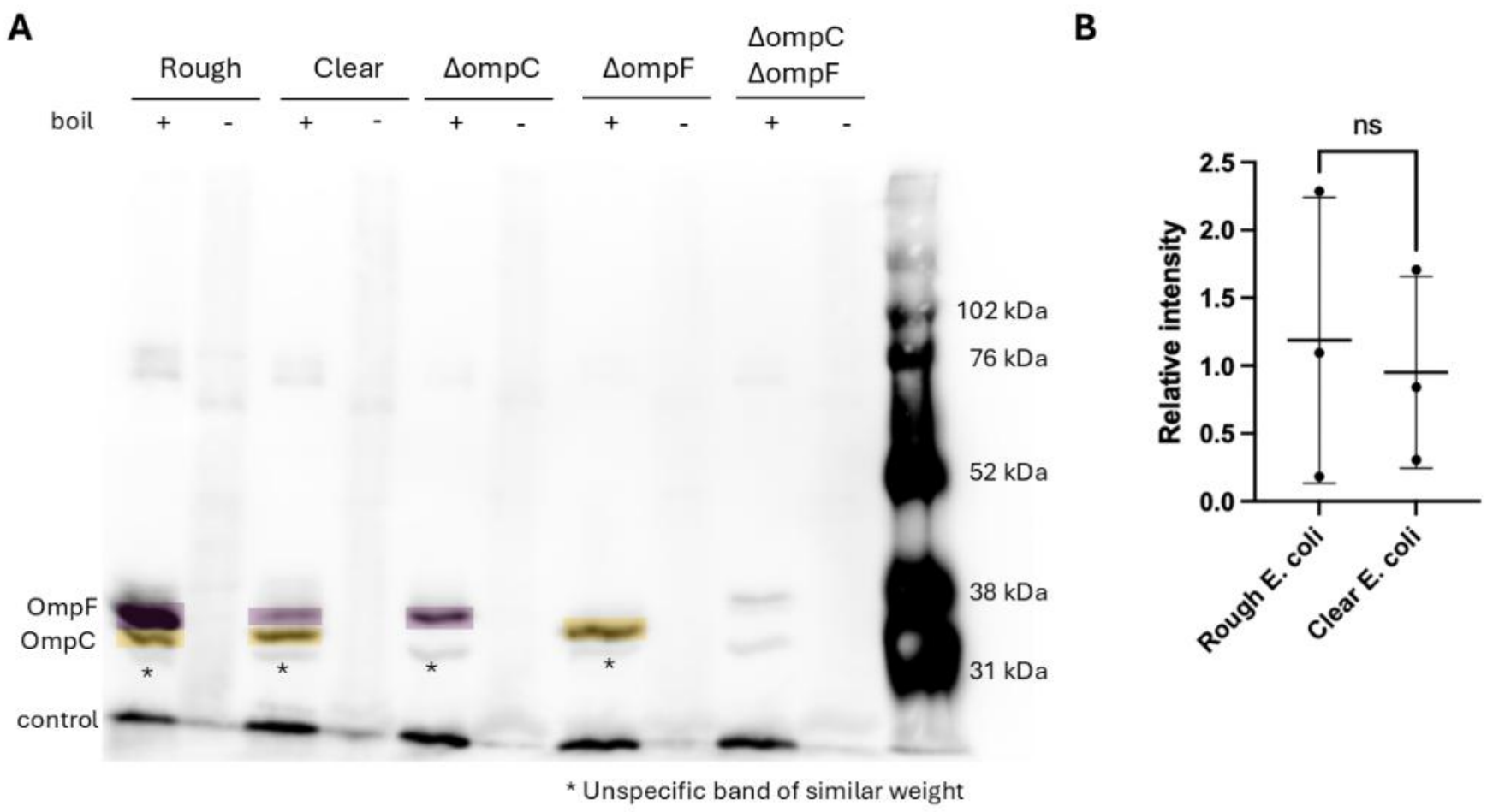

Supplementary Fig. 4. OmpC level in clear *E. coli* is comparable to that of rough *E. coli*. (A) Whole Western blot of outer membrane fraction with purple boxes highlighting the presence of OmpF and yellow boxes for the presence of OmpC. (B) Relative intensity of the OmpC band in the Western blot, normalized to the control bands at the bottom of the blot. GraphPad Prism 10 was used to analyze the data. T-test results, mean and s.d. were plotted in the same software. Source data are provided as a Source Data file.

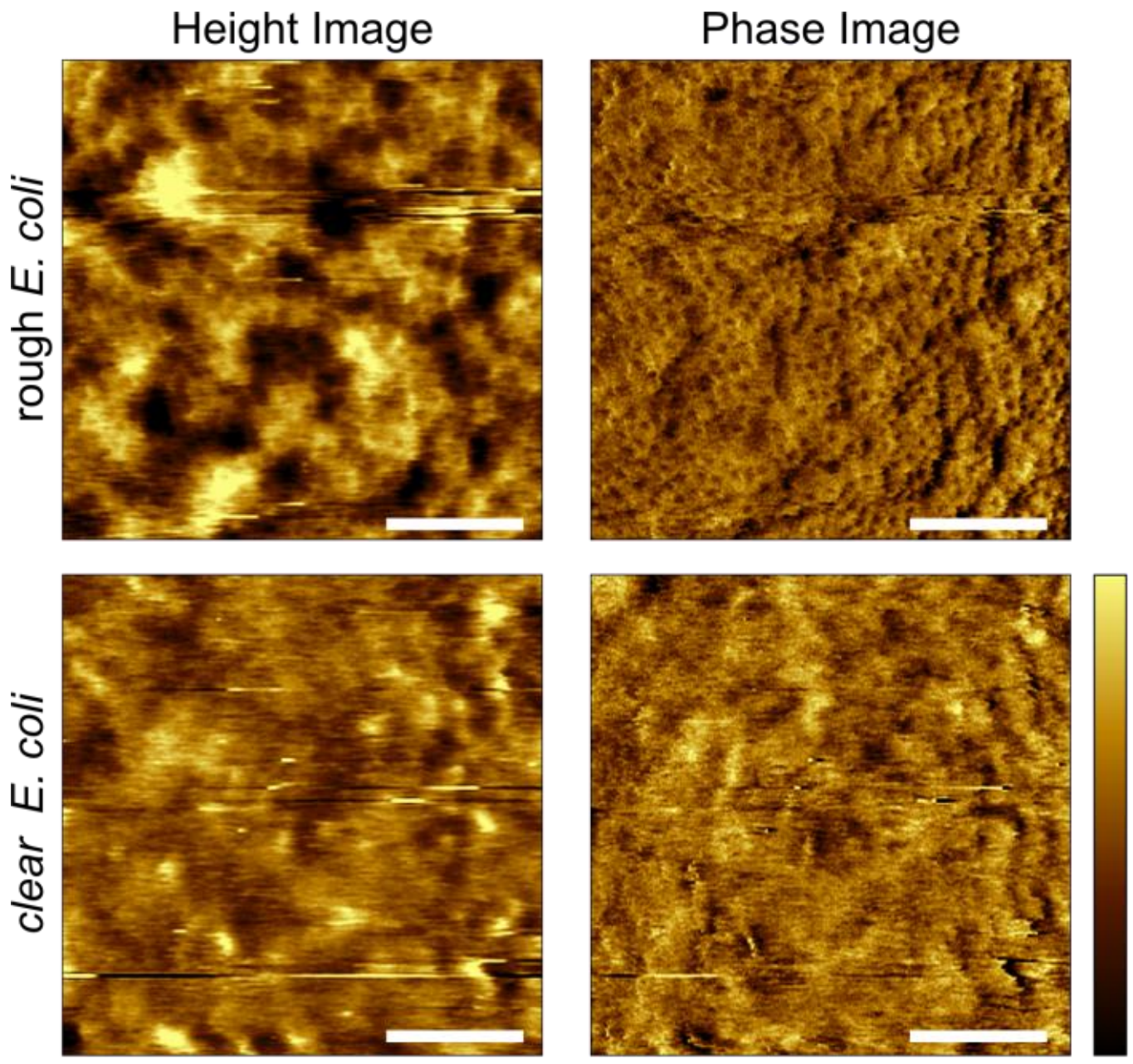

Supplementary Fig. 5. AFM height and phase images of fixed rough and clear *E. coli* cells. Chemically fixed cultures of rough and clear *E. coli* were immobilized and imaged on 2% glutaraldehyde coated glass coverslips in PHEM buffer. Consistent with the live cell data in Fig. 3, the fixed rough *E. coli* shows the porous networks associated with outer membrane protein trimers, whereas the fixed clear *E. coli* does not (*n*=2). Scale bars: 100 nm. Color (height/ phase) scales: 5 nm/ 2 deg.

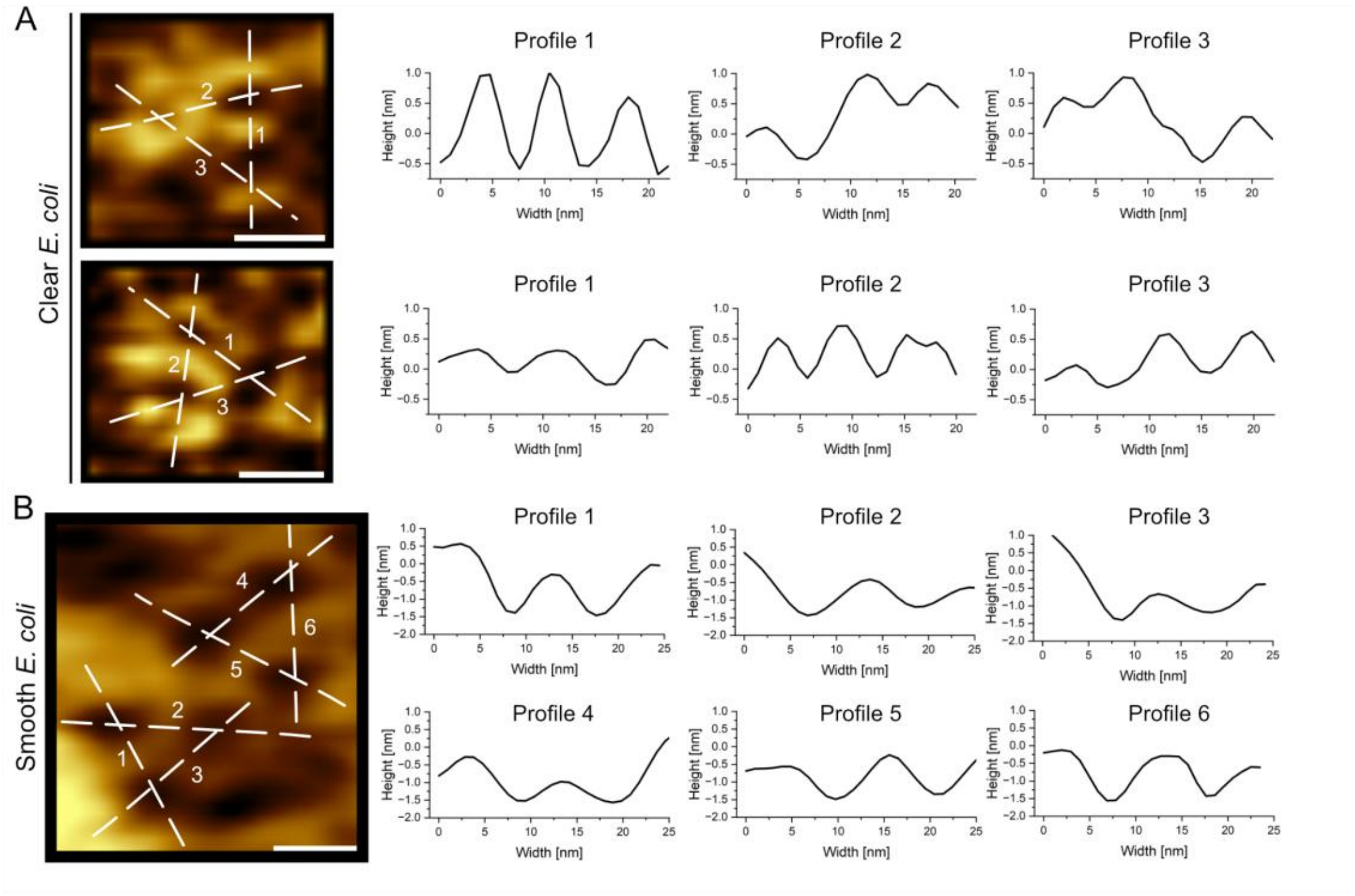

Supplementary Fig. 6. AFM height images and corresponding profiles of features observed in clear (A) and rough (B) *E. coli* outer membranes likely corresponding to higher-order assemblies of protein timers. Two different areas on the same cell are shown for clear *E. coli*. Source data are provided as a Source Data file. Scale bars: 10 nm. Color (height) scales: 2.5 nm (A, top); 1.5 nm (A, bottom); 3.5 nm (B).

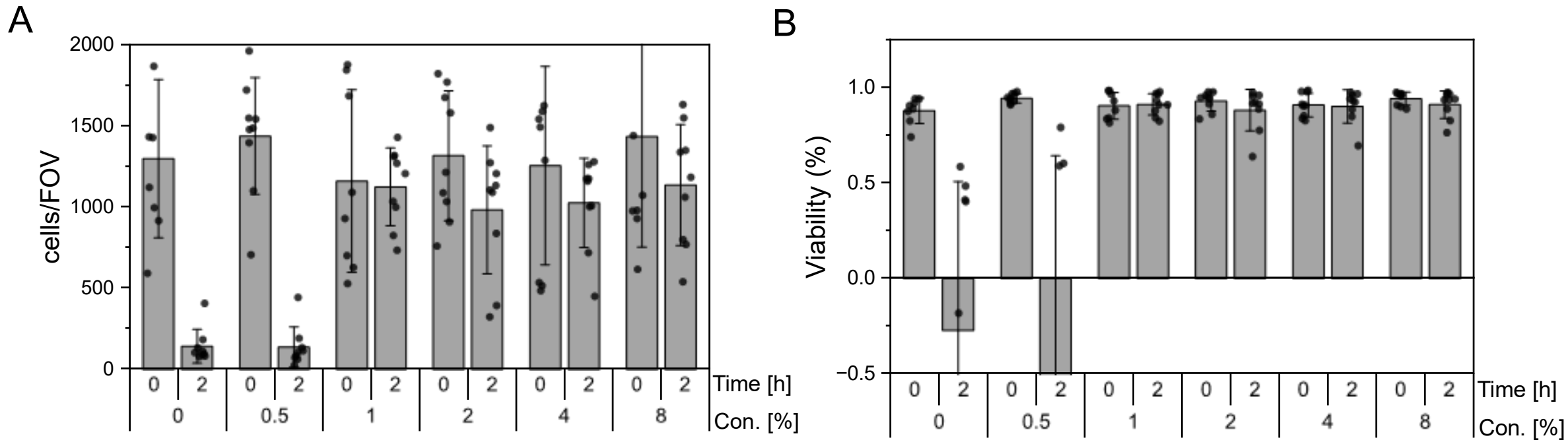

Supplementary Fig. 7. Stable P. aeruginosa PAO1 attachment is achieved on glutaraldehyde coating concentrations of 1% and above. PAO1 cells were regrown from an overnight culture in Casamino acid medium to exponential phase (OD 0.5-1). Cells were washed twice in MM and once in HEPES, as described in the Methods section, before 200 µL of bacteria at OD 0.5 were immobilized on slides coated with 0, 0.5, 1, 2, 4, or 8% glutaraldehyde for 20 min at room temperature. Cell attachment and SYTOX positivity were analyzed by optical imaging immediately after immobilization and again 2 h post-attachment following repeated surface rinsing. (A) Quantification of surface immobilization determined from bright-field images and expressed as cells per FOV, where the FOV measured 273 × 204 µm^2. (B) Cell viability, expressed as the percentage of cells maintaining cell envelope integrity (SYTOX-negative), for bacteria adhering to coated coverslips immediately after immobilization (0 h) and 2 h after immobilization at room temperature with additional surface rinsing (2 h). Data were acquired from three biological replicates, each with three independent acquisition areas (*n*=9, expect for conditions 0% and 1% at 0h with *n*=8), and are presented as mean ± s.d.. Negative cell viability values were observed when more SYTOX-positive cells were detected in fluorescence images than cells identified in the corresponding bright-field images using the Fiji Find Maxima tool. The reduced contrast in bright-field images is caused by cell lysis, which lowers the refractive index difference between the cells and the surrounding medium, thereby limiting bacterial detection[1]. Source data are provided as a Source Data file.

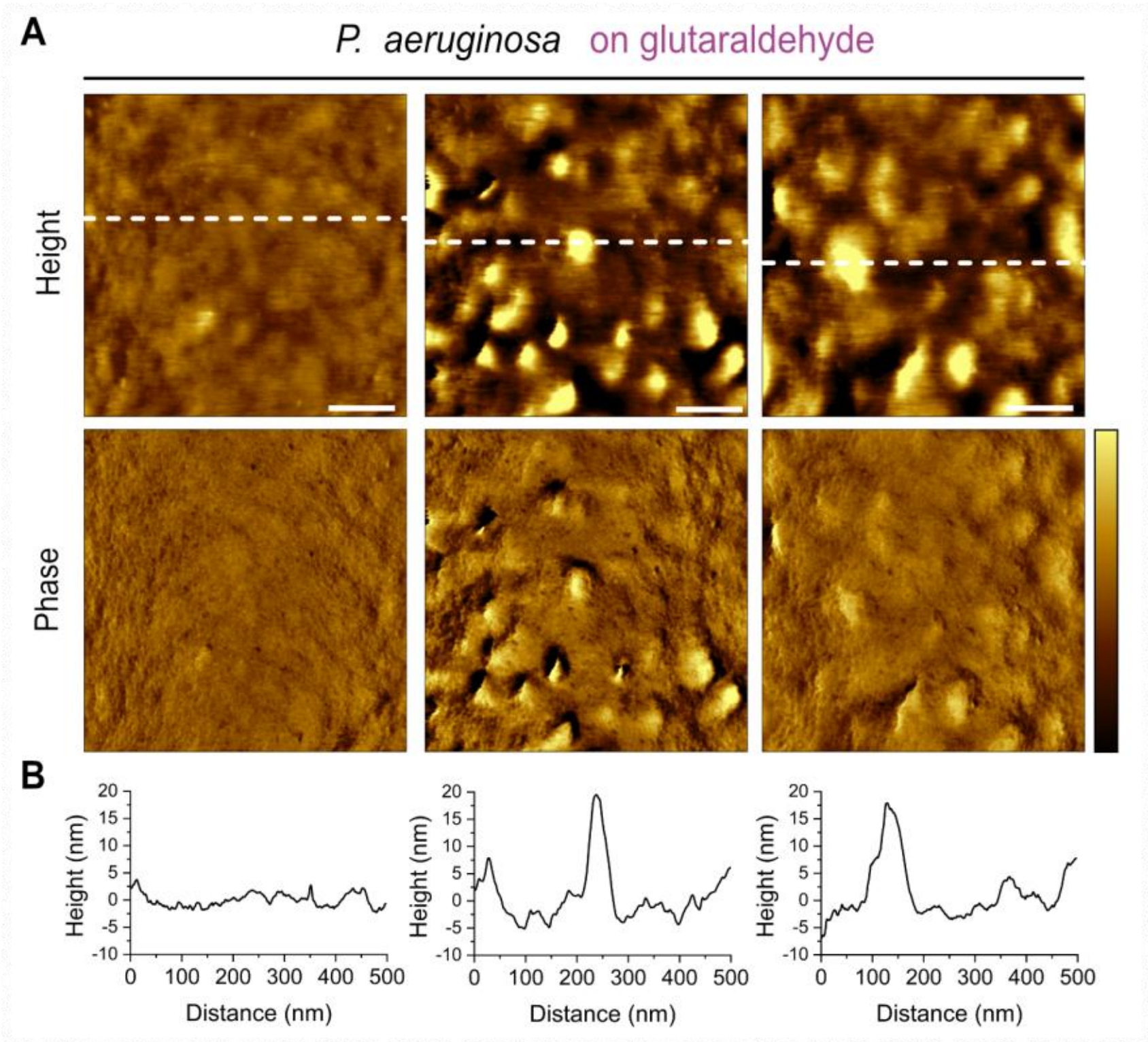

Supplementary Fig. 8. AFM characterization of *P. aeruginosa* (PAO1) cells immobilized on glutaraldehyde functionalized coverslips. A) AFM height (top row) and phase (middle row) images of three different PAO1 cells imaged on glutaraldehyde functionalized coverslips. B) Height profiles (bottom row) extracted along the white dashed lines indicated in the AFM height images in a. Source data are provided as a Source Data file. Scale bar: 100 nm. Color (height/ phase) scales: 15 nm/ 2 deg.

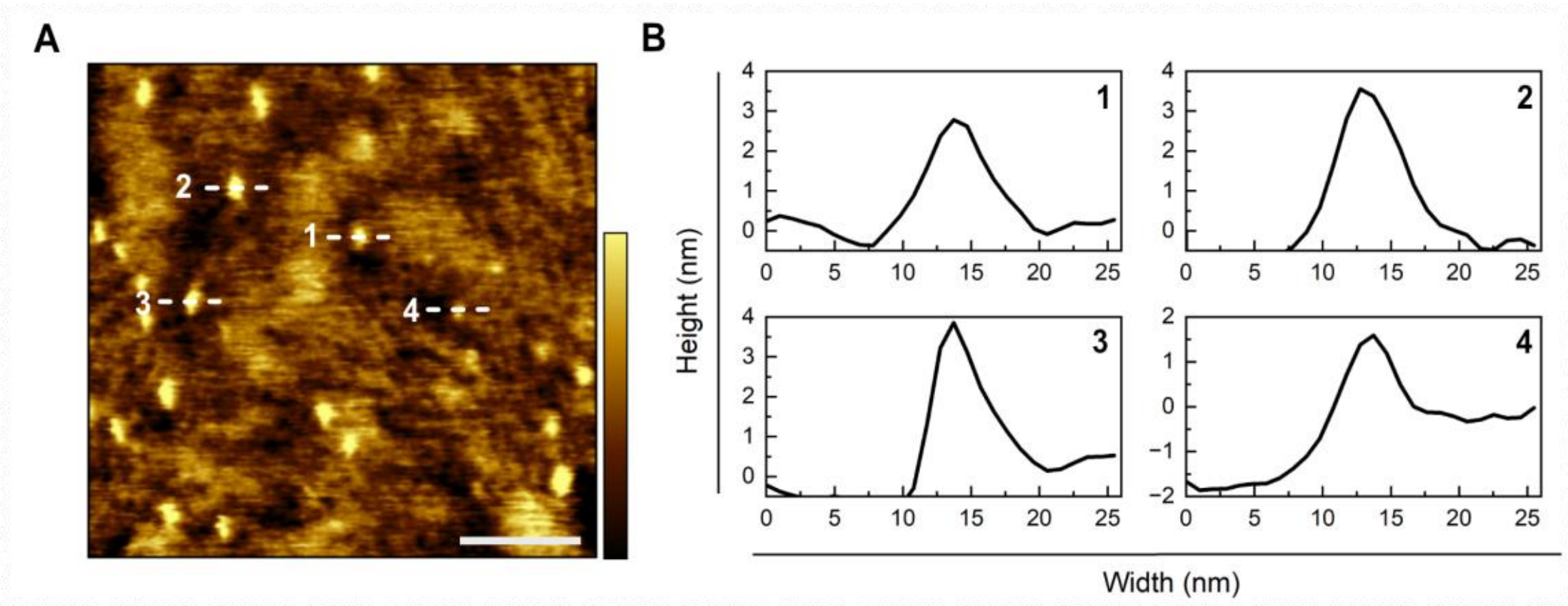

Supplementary Fig. 9. Nanosized surface protrusions of *P. aeruginosa* (PAO1). A) AFM height image of PAO1. White lines indicate the regions from which line profiles were extracted for B. B) Line profiles of four protrusions observed in a. Source data are provided as a Source Data file. Scale bar: 50 nm. Color (height) scale: 4 nm.

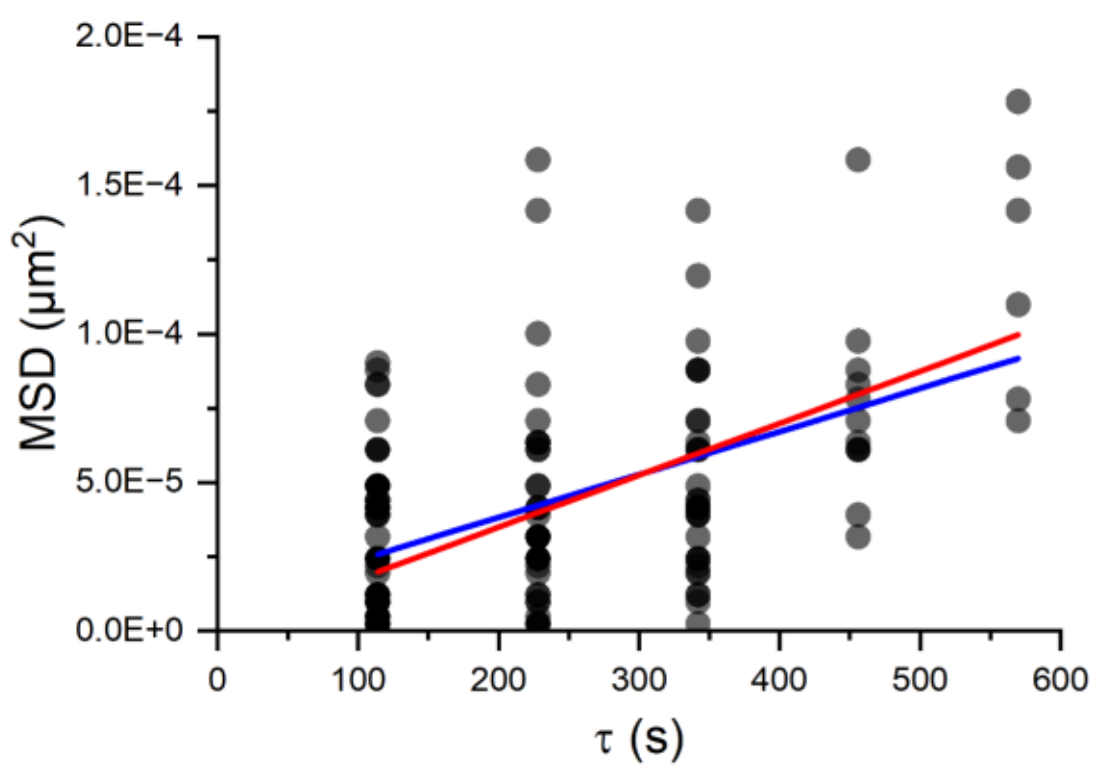

Equation	y = a + b*x
Plot	msd_um2
Weight	No Weighting
Intercept	0 ± --
Slope	1.74771E-7 ± 9.45786E-9
Residual Sum of Squares	1.48617E-7
Pearson's r	0.83439
R-Square (COD)	0.69621
Adj. R-Square	0.69417

Equation	y = a + b*x
Plot	msd_um2
Weight	No Weighting
Intercept	9.38101E-6 ± 5.4107E-6
Slope	1.44464E-7 ± 1.9845E-8
Residual Sum of Squares	1.45659E-7
Pearson's r	0.51347
R-Square (COD)	0.26366
Adj. R-Square	0.25868

Supplementary Fig. 10. Mean square diffusion versus time-lag plot and linear fits to determine the diffusion coefficient of protrusions imaged on *P. aeruginosa* (PAO1). Red line shows the linear fit with interception through (0,0), blue line shows an unconstrained linear fit. Line fit results for the constrained (top) and unconstrained linear fits (bottom) are shown to the right of the plot (*n*=1). Source data are provided as a Source Data file.

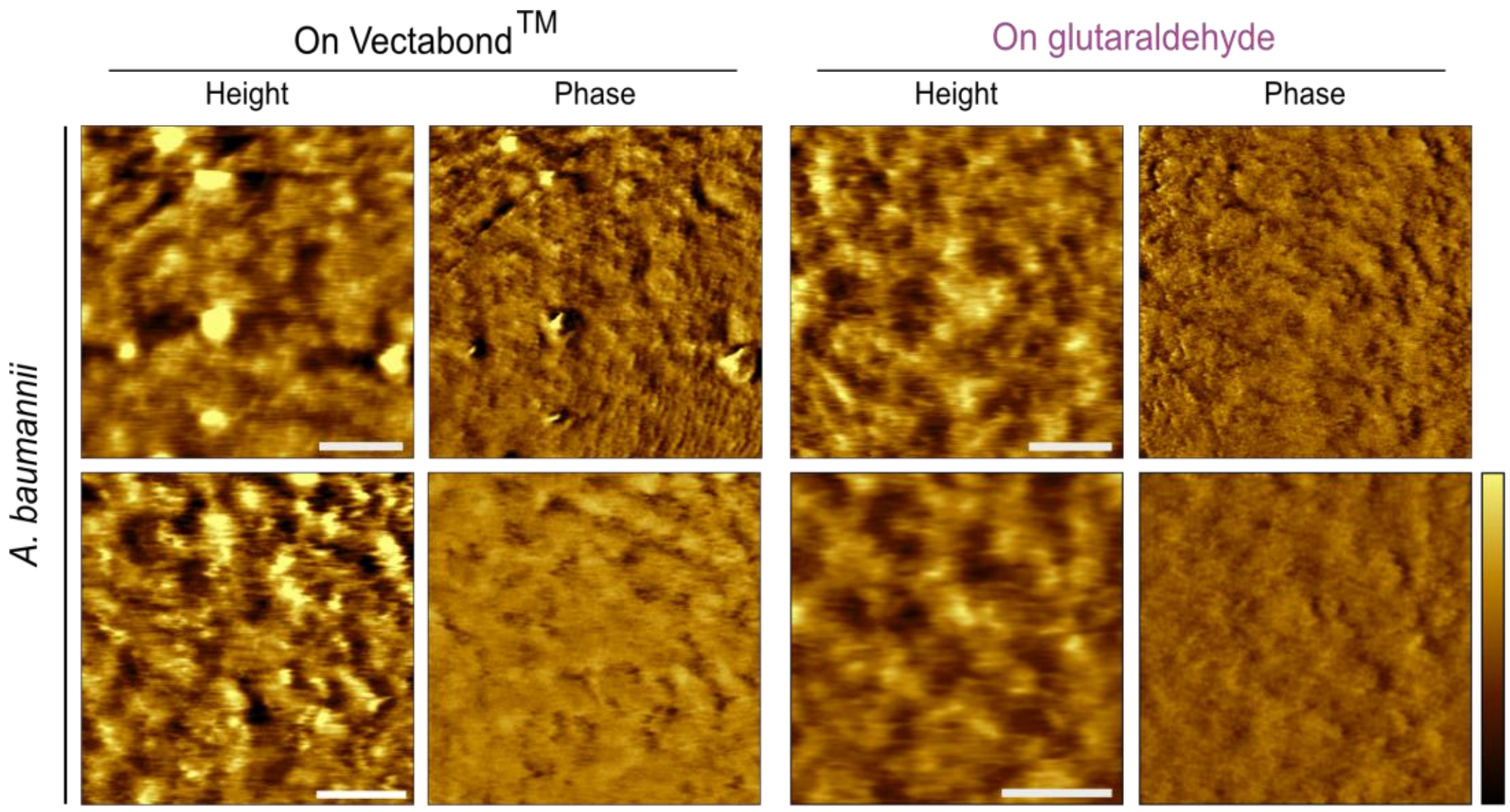

Supplementary Fig. 11. AFM height and phase images of different *A. baumannii* AB5075^{ptk} immobilized on Vectabond^{TM} (left) and glutaraldehyde (right) functionalized coverslips. Images on two independent cells are shown for both substrates. Scale bars: 100 nm. Color (height/ phase) scales: 6 nm/ 4 deg (on Vectabond^{TM}); 6 nm/ 2 deg (on glutaraldehyde).

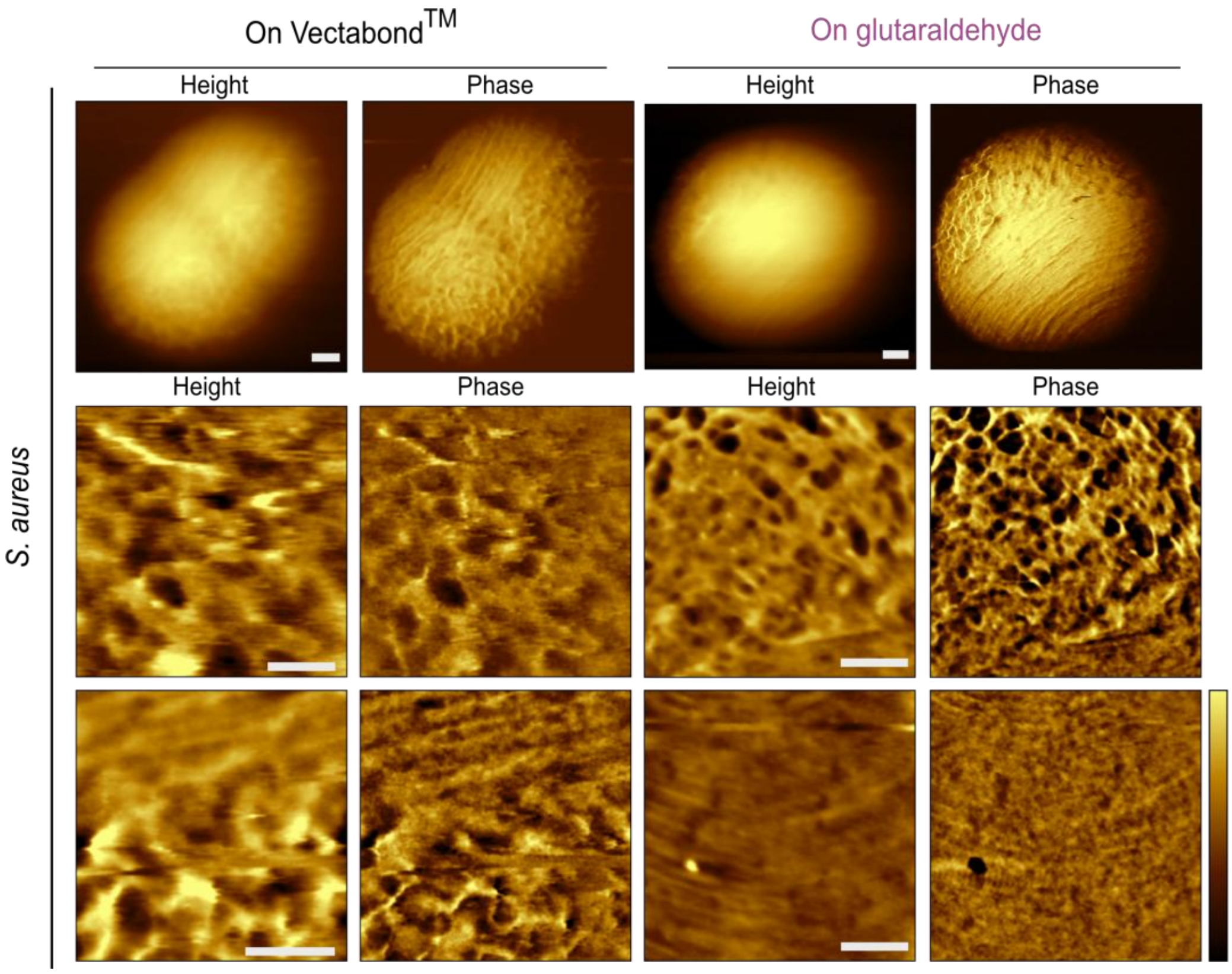

Supplementary Fig. 12. AFM height and phase images of *S. aureus* ATCC 29213 immobilized on coverslips functionalized with Vectabond™ (left) and with glutaraldehyde (right). Higher magnification images of two independent cells are shown for both substrates. Scale bars: 100 nm. Color (height/ phase) scales: 200 nm/ 12 deg (whole cells); 10 nm/ 2 deg (higher magnifications).

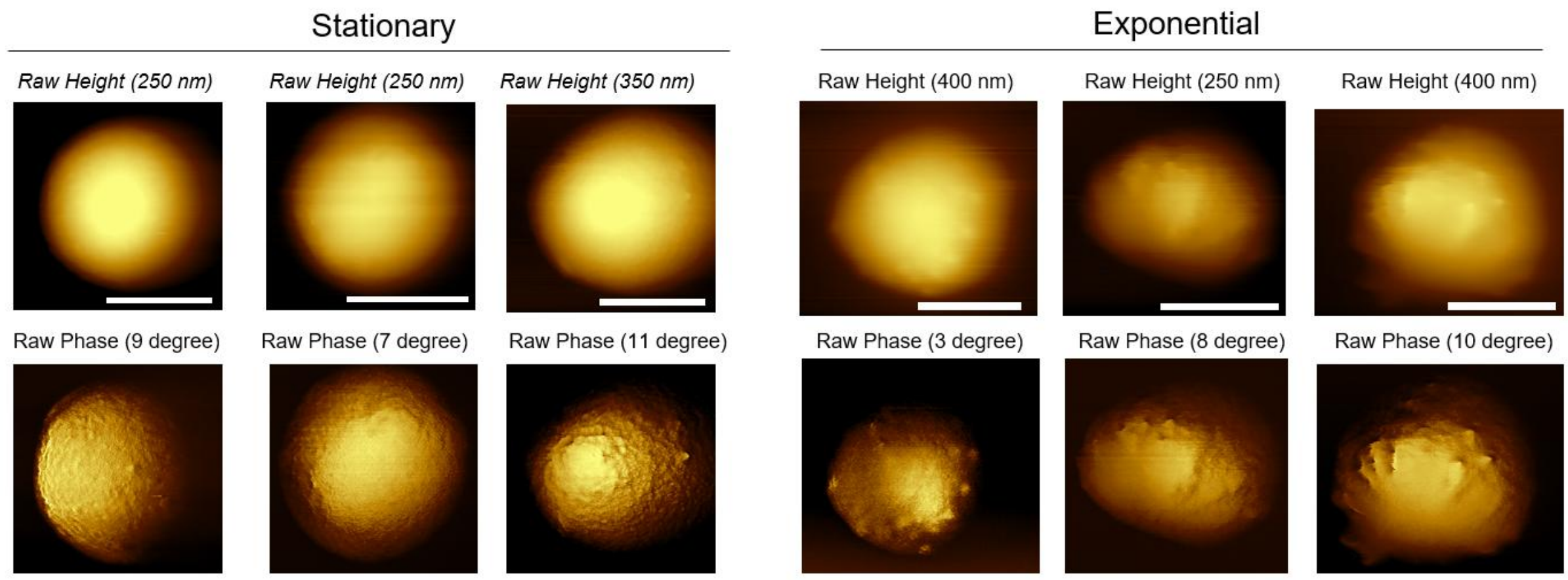

Supplementary Fig. 13. AFM height and phase images of three different bacterial pole regions in stationary (left) and mid-logarithmic growth stage (right). Images on three independent cells are shown for both growth stages. Scale bars: 500 nm.

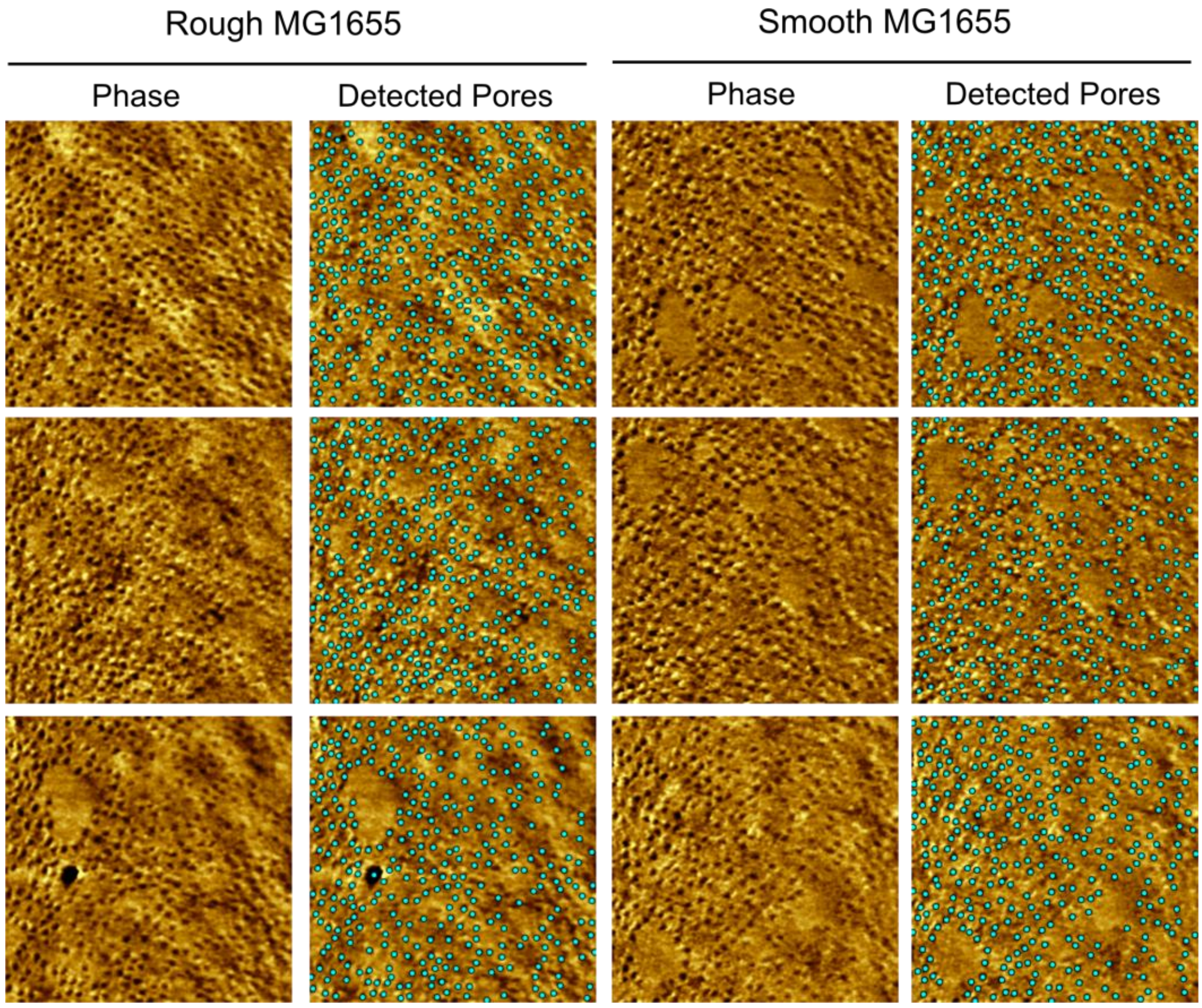

Supplementary Fig. 14. Example AFM phase images and the corresponding pore detection using Fiji. Images are of three different cells within one biological culture of rough and smooth MG1655. The center of the detected pores in the “Detected Pores” images are shown as cyan dots on the phase image. Images size: 293 nm × 293 nm.

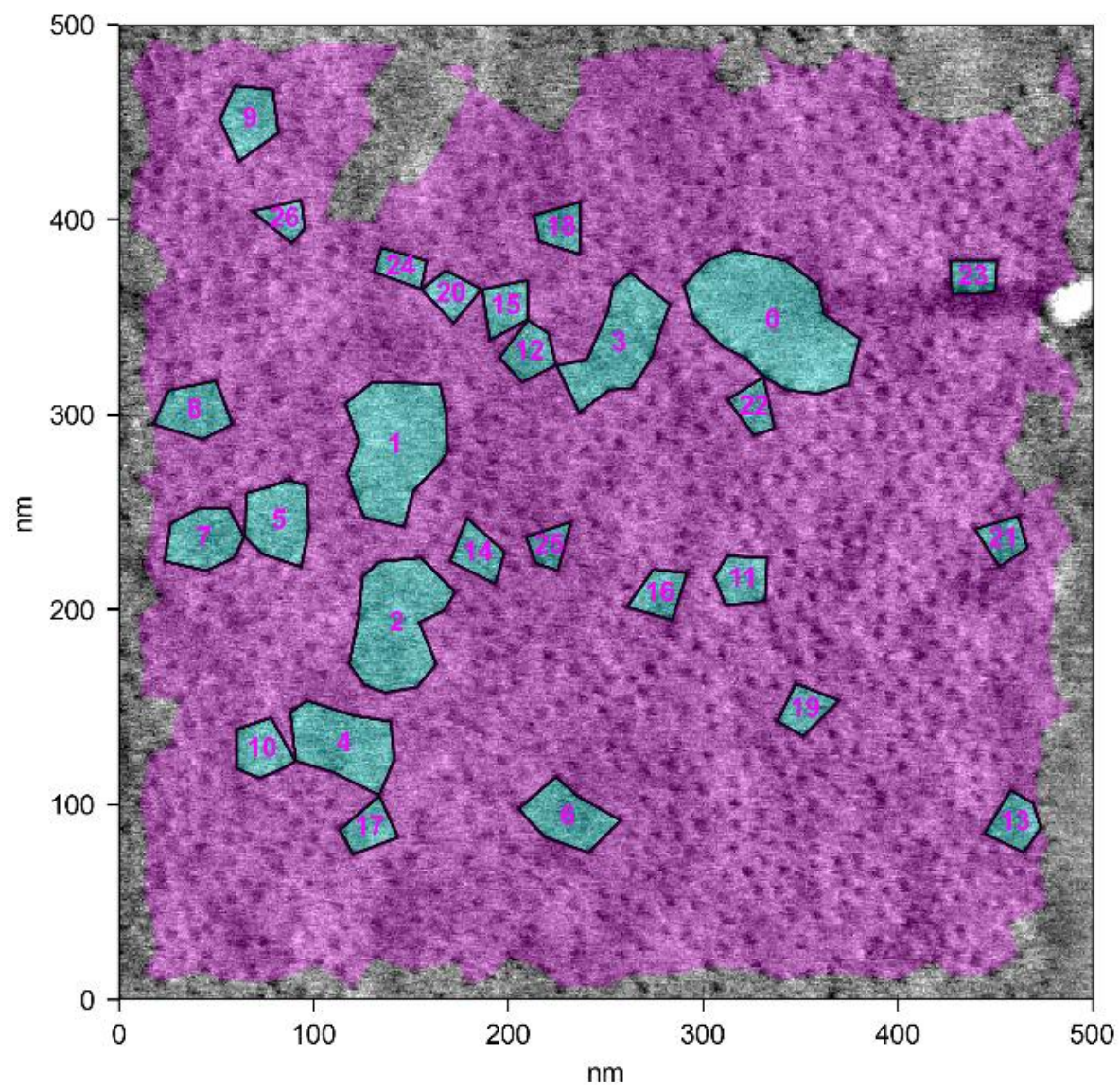

Supplementary Fig. 15. Example patch detection output file highlighting patches in cyan on a magenta background.

References:

1. Tartari, E. *et al.* Monitoring of Single-Cell Bacterial Lysis by Phages Within Integrated Optical Traps. *Adv. Opt. Mater.* 13, 2402586 (2025).
