# Supplementary material for: Nanometer-resolution imaging of living bacteria across species and cellular orientations
Source: Nat Commun. 2026 Nov 9;17:11329. doi: 10.1038/s41467-026-78975-y (PMC13748617; doi:10.1038/s41467-026-78975-y)
Supplement: Supplementary file 2 [file 41467_2026_78975_MOESM2_ESM.pdf]

Description of Additional Supplementary Files:

Supplementary Movie 1: Video displaying the dislodging of a pole-immobilized rough MG1655 cell using an AFM cantilever tip. Video shows perpendicular immobilized bacterium subjected to the AFM cantilever movement typically used for imaging. The cantilever moves vertically and upon contacting the perpendicular immobilized cell dislodges it, revealing the rod shape of the bacterium.
