# Supplementary material for: Nanometer-resolution imaging of living bacteria across species and cellular orientations
Source: Nat Commun. 2026 Nov 9;17:11329. doi: 10.1038/s41467-026-78975-y (PMC13748617; doi:10.1038/s41467-026-78975-y)
Supplement: Supplementary file 3 [file 41467_2026_78975_MOESM3_ESM.pdf]

nature portfolio

Corresponding author(s): Anna Scheeder and Bart W. Hoogenboom

Last updated by author(s): Sep 24, 2026

Reporting Summary

Nature Portfolio wishes to improve the reproducibility of the work that we publish. This form provides structure for consistency and transparency in reporting. For further information on Nature Portfolio policies, see our Editorial Policies and the Editorial Policy Checklist.

Statistics

For all statistical analyses, confirm that the following items are present in the figure legend, table legend, main text, or Methods section.

n/a	Confirmed	
☐	☒	The exact sample size (*n*) for each experimental group/condition, given as a discrete number and unit of measurement
☐	☒	A statement on whether measurements were taken from distinct samples or whether the same sample was measured repeatedly
☐	☒	The statistical test(s) used AND whether they are one- or two-sided<br>*Only common tests should be described solely by name; describe more complex techniques in the Methods section.*
☒	☐	A description of all covariates tested
☐	☒	A description of any assumptions or corrections, such as tests of normality and adjustment for multiple comparisons
☐	☒	A full description of the statistical parameters including central tendency (e.g. means) or other basic estimates (e.g. regression coefficient) AND variation (e.g. standard deviation) or associated estimates of uncertainty (e.g. confidence intervals)
☐	☒	For null hypothesis testing, the test statistic (e.g. *F*, *t*, *r*) with confidence intervals, effect sizes, degrees of freedom and *P* value noted<br>*Give P values as exact values whenever suitable.*
☒	☐	For Bayesian analysis, information on the choice of priors and Markov chain Monte Carlo settings
☒	☐	For hierarchical and complex designs, identification of the appropriate level for tests and full reporting of outcomes
☒	☐	Estimates of effect sizes (e.g. Cohen's *d*, Pearson's *r*), indicating how they were calculated

Our web collection on statistics for biologists contains articles on many of the points above.

Software and code

Policy information about availability of computer code

Data collection	ImageJ 1.54p was used for densitometry analysis of western blots and to determine the diffusion coefficient from AFM height images. A custom written code “porin_patch_analysis.py” for the quantification of the patch locations and shape characteristics can be found in the GitHub repository: https://github.com/hoogenboom-lab/bacteria-imaging.
Data analysis	Statistical analysis was performed using Origin 2024b and GraphPad Prism 10. AFM image analysis was performed with Gwyddion 2.65 and ImageJ 1.54p.

For manuscripts utilizing custom algorithms or software that are central to the research but not yet described in published literature, software must be made available to editors and reviewers. We strongly encourage code deposition in a community repository (e.g. GitHub). See the Nature Portfolio guidelines for submitting code & software for further information.

Data

Policy information about availability of data

All manuscripts must include a data availability statement. This statement should provide the following information, where applicable:

- Accession codes, unique identifiers, or web links for publicly available datasets
- A description of any restrictions on data availability
- For clinical datasets or third party data, please ensure that the statement adheres to our policy

The data generated in this study have been deposited in the UCL Research Data Repository database under accession code 33416257 [https://doi.org/10.5522/04/33416257]. Source data are provided with this paper.

Research involving human participants, their data, or biological material

Policy information about studies with human participants or human data. See also policy information about sex, gender (identity/presentation), and sexual orientation and race, ethnicity and racism.

Reporting on sex and gender	N/A
Reporting on race, ethnicity, or other socially relevant groupings	N/A
Population characteristics	N/A
Recruitment	N/A
Ethics oversight	N/A

Note that full information on the approval of the study protocol must also be provided in the manuscript.

Field-specific reporting

Please select the one below that is the best fit for your research. If you are not sure, read the appropriate sections before making your selection.

[x] Life sciences [ ] Behavioural & social sciences [ ] Ecological, evolutionary & environmental sciences

For a reference copy of the document with all sections, see nature.com/documents/nr-reporting-summary-flat.pdf

Life sciences study design

All studies must disclose on these points even when the disclosure is negative.

Sample size	Sample sizes were based on previous experiments or pilot assays that confirmed the number of replicates used provided appropriate statistical power. Unless stated, all experiments included 3 independent assays, which is sufficient for statistical analysis.
Data exclusions	Extracted AFM patch analysis data were excluded if: (1) the patches extended beyond the image boundaries, preventing their full size from being measured; (2) the patch surface area was less than 1500 nm^2, which we considered too small to be classified as patches; and (3) visual inspection revealed porin-like features within the patch area, indicating that the patches were not porin-free. These exclusion criteria are stated in the methods section.
Replication	Except where stated, all experiments were run using 3 independent assays.
Randomization	No randomization was used as measurements were not subjective.
Blinding	No blinding was used as measurements were not subjective.

Reporting for specific materials, systems and methods

We require information from authors about some types of materials, experimental systems and methods used in many studies. Here, indicate whether each material, system or method listed is relevant to your study. If you are not sure if a list item applies to your research, read the appropriate section before selecting a response.

Materials & experimental systems

n/a	Involved in the study	
[ ]	[x]	Antibodies
[x]	[ ]	Eukaryotic cell lines
[x]	[ ]	Palaeontology and archaeology
[x]	[ ]	Animals and other organisms
[x]	[ ]	Clinical data
[x]	[ ]	Dual use research of concern
[x]	[ ]	Plants

Methods

n/a	Involved in the study	
[x]	[ ]	ChIP-seq
[x]	[ ]	Flow cytometry
[x]	[ ]	MRI-based neuroimaging

Antibodies

Antibodies used	Polyclonal ompF Antibody (Biorbyt, #orb752372, LOT-W8537) was used at 1:1000 dilution.
Validation	Validation information can be found on the supplier's website: https://www.biorbyt.com/ompf-antibody-orb752372.html

Plants

Seed stocks	N/A
Novel plant genotypes	N/A
Authentication	N/A
