# Supplementary material for: Nanometer-resolution imaging of living bacteria across species and cellular orientations
Source: Nat Commun. 2026 Nov 9;17:11329. doi: 10.1038/s41467-026-78975-y (PMC13748617; doi:10.1038/s41467-026-78975-y)
Supplement: Supplementary file 4 [file 41467_2026_78975_MOESM5_ESM.pdf]

nature portfolio

# Peer Review File

# Nanometer-resolution imaging of living bacteria across species and cellular orientations

Corresponding Author: Dr Anna Scheeder

**This file contains all reviewer reports in order by version, followed by all author rebuttals in order by version.**

Version 0:

Reviewer comments:

Reviewer #1

(Remarks to the Author)
Atomic force microscopy (AFM) is a powerful technique for obtaining high-resolution images of molecular structures on the surfaces of living bacterial cells. In this manuscript, Scheeder et al. established a method for immobilizing bacterial cells on glutaraldehyde-functionalized glass surfaces through reversible covalent attachment, adapting previously reported protocols for AFM imaging of bacterial cells. Using this approach, the authors successfully visualized porin clusters and lipopolysaccharide (LPS)-enriched domains in rough, smooth, and clear Escherichia coli strains and evaluated the influence of LPS composition on AFM imaging of the E. coli cell surface.
The authors further demonstrated that glutaraldehyde-functionalized substrates can be applied to AFM imaging of Pseudomonas aeruginosa, Acinetobacter baumannii, and Staphylococcus aureus. Particularly interesting is their successful visualization of surface structures at the poles of E. coli cells, which have previously been difficult to access by AFM. Overall, the manuscript is well written and makes an important contribution to the development of robust live-cell AFM methods for molecular-level imaging of bacterial cell surfaces. Such methodologies will be valuable for advancing nanoscale microbiological studies. I recommend publication after the authors have adequately addressed the following points and the comments annotated in the manuscript.

1. The comparison of outer-membrane surface structures among the rough, smooth, and clear E. coli strains is interesting. The patch-like regions in the AFM images appear relatively flat, particularly in Fig. 3A and 3H. Are proteinaceous structures, such as porins, present within these patch regions?
If the patch regions exhibit high membrane fluidity, molecules within these regions may diffuse rapidly during image acquisition and therefore may not be clearly resolved by AFM. The authors state in the Discussion that the acquisition time for each AFM image was approximately 3 min (line 391). This relatively slow scanning speed may affect the visualization of mobile molecules in the outer membrane. If the authors consider this explanation plausible, this possibility should be discussed in the text.
In addition, the acquisition time for each complete AFM image is not reported in the Methods section or figure legends. I strongly recommend that the acquisition time for each AFM image be provided in the corresponding figure legend

2. Lines 370–376: As discussed by the authors, increased outer-membrane fluidity in the clear E. coli strain, resulting from the absence of the LPS polysaccharide portion, is one possible explanation for the reduced resolution of cell-surface imaging. Could the authors analyze and compare the diffusion coefficients of molecules on the surfaces of the clear and smooth E. coli strains? Such an analysis would provide direct evidence supporting the proposed relationship between membrane fluidity and imaging resolution.

3. Lines 331–336: During AFM imaging of bacterial cell poles, were any flagellum-associated structures, such as the flagellar hook or basal-body components, observed? Direct visualization of the flagellar motor or its associated structures could provide novel insights into the assembly and function of this supramolecular complex on the bacterial cell surface.

4. Fig. 3H: For the line-profile analyses, I recommend indicating the boundaries of the patch regions directly on the profiles. This would make the relationship between the AFM images and the corresponding height profiles easier to understand.

5. Please define the abbreviation “FOV” at its first occurrence.

(Remarks on code availability)

Reviewer #2

(Remarks to the Author)
The authors present a manuscript describing a comparative study for how to covalently adhere bacteria to a coverslip surface well enough to enable live-cell time-lapse AFM imaging. Time-lapse AFM imaging of bacteria has been considerably constrained by this technical challenge. The authors report that a glutaraldehyde treatment of a coverslip surface enables sufficient adsorbance of bacteria to enable AFM imaging of living cells. The authors subsequently demonstrate how this immobilization strategy enables for the resolution of nano-scale surface features, such as the porous network, reminiscent of outer membrane protein trimers characterized in previous studies. They equally report the presence of clear patches, which they assert are void of proteins.

The authors equally report on the extent to which the glutaraldehyde-functionalized coverslip can be used to immobilize evolutionarily disparate bacteria and how well the immobilization enables for the characterization of nanoscale surface features. The authors equally assess the quality of the covalent immobilization for rod-shaped bacteria that would be presumably immobilized at one pole and oriented such that the other pole is upright and can be scanned by AFM.

For the most part, the authors present a manuscript for which the experiments corroborate claims made. Nevertheless, some assertions seem strong and could either use further experimental evidence or be tempered. The manuscript would be no less valid or impactful in its main purpose if some assertions are to be carefully revised. One example includes the assertion made throughout the manuscript that the pore network observed on cells are outer membrane protein trimers (OmpF and OmpC). While in Benn et al. 2021 OmpF trimers were labeled with a Colicin N1-185 - mCherry fusion, and the pores observed here are reminiscent of these features, the authors assert that these observed features are the same without molecular labeling. Confirmation of expression of OmpF and OmpC by Western blot is not sufficient to note that those pore structures are these exact proteins without molecular tagging. Molecular labeling of OmpF might be technically challenging, so it might be worth tempering the assertions made throughout the manuscript. Wording on lines 223-224 represents a nice example of how to describe the architecture observed at the surface of the cells: “appeared similar in size and appearance as trimers of OmpF/OmpC”. By AFM, it is apparent that the porin network is reminiscent of these features and it perhaps suffices to allude to them as such without asserting that they are indeed all and only these Omp porins.

Figure legends especially though not exclusively in the supplementary material could use more descriptions of the figures depicted. Some typos were found (i.e. line 334).

I find this work exciting for the potential to enable a large number of fundamental microbiology studies that may follow. Phenomenological studies of these sorts of bacteria made adherent for AFM imaging are necessary to expand our fundamental understanding for how and why bacteria behave the way they do; particularly for processes and conditions for which molecular mechanisms of control remain unknown and measurable phenotypes limited by other techniques that do not provide the spatial resolution of AFM while enabling temporal dynamics. I find this work to be necessary and important and could open the potential for unexpected discoveries in the future by the authors and others alike.

Critiques:

1) The quantification of trimers is an admirable way to describe the porous network at the cell surface, particularly for bacterial surfaces that are non-homogenous and varying in architecture and molecular content. Nevertheless, it would help to have a more detailed understanding of how and why trimers are qualified as such from the images of AFM height and phase channels.
In figure 3g, for example, it is rather difficult to understand how the trimers are qualified, whereas for figure s4a the pore network is apparent. However, how does the image analysis qualify each individual trimer within a lattice of trimers? For the figure 3g trace and retrace images, are these simply five different fields where trimers are supposedly observed on a clear MG1655 cell or is this a time-lapse?

2) An assertion is made in multiple places that “trimer-free patches” (line 189) are “devoid of trimeric outer membrane proteins” (line 208). I think the authors mean “void”, but my English might not be up to par. Nevertheless, the assertion is rather strong. These areas seem void of the porous network reminiscent of trimeric outer membrane proteins. You do not molecularly label these zones to know whether they are or are not protein-free, even though they may very well be. The colicin tag could solve this, but again that might be a technically challenging and time-consuming experiment. It may very well suffice to say that there are smooth patches void of the pore network reminiscent of trimeric outer membrane proteins. If there are or are not proteins located there that the authors may or may not be able to resolve by AFM does not retract the validity of the work in this manuscript.

3) Line 164, the authors aimed to resolve outer membrane porin structures reminiscent of protein trimers (OmpF and OmpC).

4) Line 179-181, sentence could use a reference.

5) Paragraph 212-22: A bar chart or some other quantitative representation alongside the Western blot would be helpful to

corroborate the claim made that OmpC levels are similar between clear and rough strains of E. coli. The difference in staining by Western blot for OmpF is apparent by eye; the difference or similarity in OmpC expression is slight, but if one corrects for the control band (which is not mentioned in the figure legends), it could very well be that the difference in OmpC expression is statistically significant. This does not change the main conclusions of the manuscript, but the accuracy of the assertion made in the results is important.

6) For Figure 3E, why not show phase images, which tend to have greater contrast than the height channel? Would we be able to resolve the absence of surface pores easier?

7) For figure S3. Mention in the figure legend what the boil denotes. It is mentioned on line 557 that half of the sample was boiled presumably to liberate proteins from the outer membrane sample. I expect that the control band is at the bottom of each boiled lane in the Western blot. Mention in the figure legend that it is the control and mention the antibody used and protein labeled in the methods section. If there is no control, then I am afraid that comparisons between samples cannot be made, only bands within each sample.
Were the blots only labeled with anti-OmpF? Is this a monoclonal or polyclonal antibody that also labels OmpC or is OmpC naturally labelled by this antibody. Was this antibody procured or harvested specifically for this study from rabbits, I presume?

8) On line 256 it is mentioned that glutaraldehyde exhibits a 2- to 5-fold higher number of cells immobilized of (A. baumannii and S. aureus). Where is the quantification of this observation?

9) The authors mention on lines 461-462 that they used a 2% glutaraldehyde solution for functionalizing the coverslip surface. Is this optimal? Is it possible to quantitate a coating efficiency per surface area? Comparisons are made for how well glutaraldehyde functionalization enables bacterial immobilization. Some sort of quantification for how many bacteria (of specific strains or species) adsorb on the coverslip surface functionalized with specific concentrations of glutaraldehyde solution would be helpful for qualifying how impactful the comparisons are made by the authors. This is perhaps pertinent for understanding how and why rod-shaped bacilli can be found adherent in an upright orientation that is favorable to image polar regions (lines 318-322).

10) Functionalization with lectins is intriguing as well for the fundamental biology that can be revealed for individual bacteria. However, lectins often become covered in gunk and their binding affinity becomes attenuated. I imagine that glutaraldehyde and perhaps even Vectabond also age at certain rates for these sorts of experiments. It is commendable that the authors show a quantification of bacterial immobilization after 2 hours. This may represent a few generations of the cell cycle for bacteria grown at their “optimal” lab culture conditions. Figure 2e provides some indication that cells can divide. This immobilization strategy is relevant for studying fundamental cell processes and features on a short-midterm timescale, which is commendable in itself. Is that the extent of it, or can this study already project further to studying more long-term timescale cell processes relevant to pathogens (though perhaps in non-pathogenic models) like stress tolerance?

11) The authors use SYTOX as a proxy for cell viability, as a commonly used indicator for “living” and “dead” cells. It is not a perfect tool as bacteria do at times stain positively with SYTOX and yet remain alive. Is there any understanding as to how many cells of the same cells are tracked and considered SYTOX positive or negative, exhibit growth or not, and are or are not observed to divide within a 2-hour window, within a field of view, in a time-lapse? This might not be technically possible in the authors’ setup if there is not temperature control and thus the time required for a cell cycle at room temperature is longer than the 2-hour long studies done or the 33-min time-lapse show in figure 2e of an E. coli cell undergoing division. Another possibility would be to track how many cells are int eh process of dividing as often in bacteria septum formation (even incomplete) is detectable.

12) Line 128-129 brings up a point that washing removes unadhered cells. It is also important to note that bacteria even within isogenic, clonal populations are phenotypically heterogeneous to various extents. It could very well be that unadhered cells are non-adherent for mechanical reasons, that they never had their chance to adsorbed within 30 min of incubation (bad luck) or that their surface physiology does not make them amenable to imaging. This should not take away from the manuscript, as now we can at least envisage imaging cells, but perhaps not all and for biological reasons.

13) In Figure 2b, attachement efficiency was quantified from brightfield images. Are these AFM-imageable bacteria? How would a quantification using brightfield compare to AFM imaging efficiency? At the end of the day, the manuscript cares about whether we can readily image adherent bacteria.

14) Phase images can offer impressive contrast as compared to height images. Is there a reason not to show phase images in figure 3e to potentially reveal the pore network in rough E. coli and its absence in the “clear” strain?

15) Figure 4d, mention in the figure legend (lines 301-302) that these images are of the height channel (I assume that is what they are). Or is this a 3D representation of height images?

16) On lines 261-262 it is mentioned that “height undulations at the nanoscale” are observable, but I don’t know what the authors refer to. Are they mentioning the seeming protrusions from the surface? An undulation is well defined in mechanics as having a rhythmic pattern of wavelength, amplitude and phase. A color bar is needed for Figure S5.

17) Lines 316-319: is there data quantifying how discrete incubation times increase the rate of attachement of MG1655 in an orientation favorable to imaging polar regions?

18) Line 335, an assertion is made that the morphology of a bacterial pole being hemispherical is an “ideal” shape and the pole of a cell harvested from exponential growth phase exhibited “morphological defects”. What is the ideal nature of the pole shape referring to? From a biological perspective, an individual bacterium that the authors can image by AFM is in a certain physiological state in stationary or exponential phase of bulk growth conditions, which may or may not be ideal or defective for any number of other reasons. E. coli has extracellular structures like fimbriae or flagella. There is no squiggle to indicate that these points in Figure 5c are long and in motion, but these “defects” might very well not be defects at all.

19) Line 369, Figures 2 and 4 are cited for presenting evidence of cell viability by SYTOX staining: okay. On line 368 it is mentioned that cells are observed to be able to grow and divide. I see evidence of division in figure 2e, though not really growth (unless a kymograph can be produced or a quantification of elongation conducted). Furthermore, I do not see where there is evidence that the other bacterial model organisms are observed to grow and divide. We at least have evidence for “viability” as measured by SYTOX and division for E. coli.

20) Lines 393-395: it is mentioned that AFM imaging of S. aureus revealed “peptidoglycan architectures as reported previously”. Again, these are likely peptidoglycan architectures, but there is not a molecular labeling of these architectural structures to indicate that there are composed of peptidoglycan. They are reminiscent of peptidoglycan structures reported previously.

(Remarks on code availability)

**Open Access** This Peer Review File is licensed under a Creative Commons Attribution 4.0 International License, which permits use, sharing, adaptation, distribution and reproduction in any medium or format, as long as you give appropriate credit to the original author(s) and the source, provide a link to the Creative Commons license, and indicate if changes were made.
In cases where reviewers are anonymous, credit should be given to 'Anonymous Referee' and the source.
The images or other third party material in this Peer Review File are included in the article’s Creative Commons license, unless indicated otherwise in a credit line to the material. If material is not included in the article’s Creative Commons license and your intended use is not permitted by statutory regulation or exceeds the permitted use, you will need to obtain permission directly from the copyright holder.
To view a copy of this license, visit https://creativecommons.org/licenses/by/4.0/

**Point-by-Point Response to Reviewers of manuscript NCOMMS-26-047292-T for Nature Communications "Nanometer-resolution imaging of living bacteria across species and cellular orientations"**

We would like to thank the editor and reviewers for the time and effort they have invested in evaluating our manuscript and we are pleased to hear the positive feedback. Below, we respond to every point raised by the reviewers, and we have revised the manuscript accordingly.

For ease of reference, the reviewer comments are reproduced in **black** and our responses are provided in **green**. Changes to the manuscript are highlighted in yellow.

---

**Reviewer #1 (Remarks to the Author):**

Atomic force microscopy (AFM) is a powerful technique for obtaining high-resolution images of molecular structures on the surfaces of living bacterial cells. In this manuscript, Scheeder et al. established a method for immobilizing bacterial cells on glutaraldehyde-functionalized glass surfaces through reversible covalent attachment, adapting previously reported protocols for AFM imaging of bacterial cells. Using this approach, the authors successfully visualized porin clusters and lipopolysaccharide (LPS)-enriched domains in rough, smooth, and clear Escherichia coli strains and evaluated the influence of LPS composition on AFM imaging of the E. coli cell surface. The authors further demonstrated that glutaraldehyde-functionalized substrates can be applied to AFM imaging of Pseudomonas aeruginosa, Acinetobacter baumannii, and Staphylococcus aureus. Particularly interesting is their successful visualization of surface structures at the poles of E. coli cells, which have previously been difficult to access by AFM.

Overall, the manuscript is well written and makes an important contribution to the development of robust live-cell AFM methods for molecular-level imaging of bacterial cell surfaces. Such methodologies will be valuable for advancing nanoscale microbiological studies. I recommend publication after the authors have adequately addressed the following points and the comments annotated in the manuscript.

We thank Reviewer #1 for their positive feedback and have addressed the raised comments point by point below.

1. The comparison of outer-membrane surface structures among the rough, smooth, and clear E. coli strains is interesting. The patch-like regions in the AFM images appear relatively flat, particularly in Fig. 3A and 3H. Are proteinaceous structures, such as porins, present within these patch regions?

We thank Reviewer #1 for their question on the presence or absence of proteinaceous structures in the observed patch-like regions. Our previously published work found that biochemically specific labels for (trimeric) OmpF and (monomeric) OmpA did not colocalise with the patch-like regions (PNAS, 2021, 10.1073/pnas.2112237118). Based on the smooth/flat appearance of the patches and the lack of colocalisation of some of the (in *E. coli)* most abundant trimeric and monomeric OMPs with the smooth patches, we therefore concluded that the patches are largely free of proteins. We have carried over that conclusion into this manuscript. Nonetheless, since the complete absence of proteins in the patch-like areas is not proven in this manuscript, we have modified the text accordingly (**lines 214-217**).

If the patch regions exhibit high membrane fluidity, molecules within these regions may diffuse rapidly during image acquisition and therefore may not be clearly resolved by AFM. The authors state in the Discussion that the acquisition time for each AFM image was approximately 3 min (line 391). This relatively slow scanning speed may affect the visualization of mobile molecules in the outer membrane. If the authors consider this explanation plausible, this possibility should be discussed in the text.

We agree with Reviewer #1 that movement within the outer membrane can affect the appearance of the AFM data. Related to this observation, we have previously shown that the removal of OmpA, a protein linking the outer membrane to the underlying peptidoglycan network, likely caused increased membrane fluidity and reduced the visibility of the trimeric porin network for AFM (PNAS, 2024, 10.1073/pnas.2416426121). In general, images will appear blurrier if objects diffuse during the acquisition period. We previously highlighted the limitations of AFM in this context in **lines 229-231** and **397-400** in the manuscript and, in response to this reviewer comment, have now further emphasised them with the addition of **lines 231-234**.

In addition, the acquisition time for each complete AFM image is not reported in the Methods section or figure legends. I strongly recommend that the acquisition time for each AFM image be provided in the corresponding figure legend.

We have followed the reviewer's recommendation to make the image acquisition times more explicit in the manuscript, in the methods section in **lines 553-554** (as an alternative for providing them in the figure legends, to avoid excessive clutter).

2. Lines 370–376: As discussed by the authors, increased outer-membrane fluidity in the clear E. coli strain, resulting from the absence of the LPS polysaccharide portion, is one possible explanation for the reduced resolution of cell-surface imaging. Could the authors analyze and compare the diffusion coefficients of molecules on the surfaces of the clear and smooth E. coli strains? Such an analysis would provide direct evidence

supporting the proposed relationship between membrane fluidity and imaging resolution.

We thank Reviewer #1 for suggesting a further exploration of outer membrane fluidity as a potential explanation for the observed reduction in contrast or resolution in the clear *E. coli* strain.

To enable a more definite conclusion on the role of membrane fluidity in this context, we chose to do additional experiments in which membrane fluidity was deliberately constrained by chemical fixation, now reported in **lines 234-236** of the results section, in the sample preparation protocol in **lines 525-531** of the methods section, and in the new **Fig. S5**. If membrane fluidity plays a role in compromising AFM resolution on the clear *E. coli*, we would expect that the resolution would be improved by chemically fixing the bacteria. To fix clear and rough *E. coli,* we followed a cryo-electron tomography fixation protocol to preserve the architecture of the bacterial envelope (Nat. Commun., 2020, 10.1038/s41467-020-19619-7).

With the caveat that fixation led too somewhat less robust imaging conditions than for the live samples, the fixed samples showed features that were fully consistent with our observations on living cells: the pore networks were readily resolved on the fixed rough *E. coli* but absent from the fixed clear *E. coli* (see new **Fig. S5**).

These new data provide further support for our interpretation of the observed difference between clear and rough/smooth *E. coli* as due to the contrast provided by the LPS oligosaccharide (which is lacking in clear *E. coli*), illustrated in (**Fig. 3e**), as opposed to an interpretation in terms of membrane fluidity.

3. Lines 331–336: During AFM imaging of bacterial cell poles, were any flagellum-associated structures, such as the flagellar hook or basal-body components, observed? Direct visualization of the flagellar motor or its associated structures could provide novel insights into the assembly and function of this supramolecular complex on the bacterial cell surface.

We thank Reviewer #1 for this very interesting question. We did not directly observe but also have not actively looked for flagella-associated features on bacterial poles. The *E. coli* MG1655 model bacterium used in this study is peritrichous, thus does not necessarily have a flagellum on the poles. We also do not routinely observe flagella associated features of exponentially grown cells along the long axis of these bacteria which may be attributed to the rigorous bacterial washing we perform pre-immobilisation, potentially causing detachment of flagella detachment and making it more difficult to localise these features. We fully agree with Reviewer #1 that it would be very intriguing to study if flagella-associated features can be observed especially on

strains with polar flagella, noting that this would likely represent an entire new study on its own.

4. Fig. 3H: For the line-profile analyses, I recommend indicating the boundaries of the patch regions directly on the profiles. This would make the relationship between the AFM images and the corresponding height profiles easier to understand.

We thank Reviewer #1 for this suggestion and have indicated the boundaries of the patch region in the line profiles to easier visualise the boundary regions. Please see **Fig. 3H** and the figure legend description in **lines 204-207**.

5. Please define the abbreviation "FOV" at its first occurrence.

We thank Reviewer #1 for this observation. We have now added the abbreviation for FOV at its first occurrence in **line 132**.

---

**Reviewer #2 (Remarks to the Author):**

The authors present a manuscript describing a comparative study for how to covalently adhere bacteria to a coverslip surface well enough to enable live-cell time-lapse AFM imaging. Time-lapse AFM imaging of bacteria has been considerably constrained by this technical challenge. The authors report that a glutaraldehyde treatment of a coverslip surface enables sufficient adsorbance of bacteria to enable AFM imaging of living cells. The authors subsequently demonstrate how this immobilization strategy enables for the resolution of nano-scale surface features, such as the porous network, reminiscent of outer membrane protein trimers characterized in previous studies. They equally report the presence of clear patches, which they assert are void of proteins.

The authors equally report on the extent to which the glutaraldehyde-functionalized coverslip can be used to immobilize evolutionarily disparate bacteria and how well the immobilization enables for the characterization of nanoscale surface features. The authors equally assess the quality of the covalent immobilization for rod-shaped bacteria that would be presumably immobilized at one pole and oriented such that the other pole is upright and can be scanned by AFM.

For the most part, the authors present a manuscript for which the experiments corroborate claims made. Nevertheless, some assertions seem strong and could either use further experimental evidence or be tempered. The manuscript would be no less valid or impactful in its main purpose if some assertions are to be carefully revised. One example includes the assertion made throughout the manuscript that the pore network

observed on cells are outer membrane protein trimers (OmpF and OmpC). While in Benn et al. 2021 OmpF trimers were labeled with a Colicin N1-185 - mCherry fusion, and the pores observed here are reminiscent of these features, the authors assert that these observed features are the same without molecular labeling. Confirmation of expression of OmpF and OmpC by Western blot is not sufficient to note that those pore structures are these exact proteins without molecular tagging. Molecular labeling of OmpF might be technically challenging, so it might be worth tempering the assertions made throughout the manuscript. Wording on lines 223-224 represents a nice example of how to describe the architecture observed at the surface of the cells: "appeared similar in size and appearance as trimers of OmpF/OmpC". By AFM, it is apparent that the porin network is reminiscent of these features and it perhaps suffices to allude to them as such without asserting that they are indeed all and only these Omp porins.

Figure legends especially though not exclusively in the supplementary material could use more descriptions of the figures depicted. Some typos were found (i.e. line 334).

I find this work exciting for the potential to enable a large number of fundamental microbiology studies that may follow. Phenomenological studies of these sorts of bacteria made adherent for AFM imaging are necessary to expand our fundamental understanding for how and why bacteria behave the way they do; particularly for processes and conditions for which molecular mechanisms of control remain unknown and measurable phenotypes limited by other techniques that do not provide the spatial resolution of AFM while enabling temporal dynamics. I find this work to be necessary and important and could open the potential for unexpected discoveries in the future by the authors and others alike.

We thank Reviewer #2 for their comments, advice, and positive feedback. We have addressed the raised points below, tempered assertions accordingly, and added more detailed descriptions to the figure legends as highlighted in the manuscript.

To address the specific point raised by Reviewer #2 on the assertion that the apparent pores are OmpF/OmpC features, we have now rephrased the text (**lines 167, 192, 213, 214-216, 356**) to make more explicit that we assume the apparent pores are the same features as observed and reported in our previously published work (PNAS, 2021, 10.1073/pnas.2112237118).

That previous work established that the observed porous network is due to the trimeric outer membrane proteins OmpF and OmpC, through analysis of local lattice dimensions of the pores, through biochemical labelling (as the reviewer suggests here) and through specific mutations (PNAS, 2021, 10.1073/pnas.2112237118). As reported in **Fig. 3a** and **Fig. 3b**, the observed porous features in this manuscript are qualitatively (overall appearance) and quantitatively (pore network dimensions and pores/area) consistent

with that previously published work on the same bacterial strain. We therefore feel confident in assuming that the observed pore features are associated with OmpF/OmpC trimers, with appropriate caveats as per the reviewer's recommendation.

We agree with Reviewer #2 that it is likely that other outer membrane proteins are embedded in the network that may remain unresolved by AFM. We previously confirmed this for the monomeric transporter FepA by specific biochemical labelling and AFM (Sci. Adv., 2022, 10.1126/sciadv.adc9566).

Critiques:

1) The quantification of trimers is an admirable way to describe the porous network at the cell surface, particularly for bacterial surfaces that are non-homogenous and varying in architecture and molecular content. Nevertheless, it would help to have a more detailed understanding of how and why trimers are qualified as such from the images of AFM height and phase channels.

In figure 3g, for example, it is rather difficult to understand how the trimers are qualified, whereas for figure s4a the pore network is apparent. However, how does the image analysis qualify each individual trimer within a lattice of trimers? For the figure 3g trace and retrace images, are these simply five different fields where trimers are supposedly observed on a clear MG1655 cell or is this a time-lapse?

We thank Reviewer #2 for the comment and highlighting that the identification of the porous network and the associated analysis were not described sufficiently well.

The porous features for rough and smooth bacteria were quantitatively assessed for **Fig. 3b**. We apologise that the methods section did not provide a sufficiently thorough explanation of how the individual pores, associated with trimeric porins, where identified. We have addressed this by revision of the methods section (**lines 559-569**) and also revised **Fig. S14** to better highlight examples of pore localisations.

Furthermore, we would like to apologise for any confusion regarding pore quantifications relating to **Fig. 3g**. **Fig. 3g** shows five different fields of views of rarer features observed on the clear *E. coli* surface, where we did not find the extensive pore networks as seen on rough and smooth *E. coli*. Although the statistics appeared insufficient for more quantitative analysis, the features appeared genuine hence we felt it appropriate to report these. We did not perform pore quantifications beyond an inspection of the dimensions of the features (**Fig. S6**). We have further revised the image caption (**line 202**) to clarify that five different fields were represented in **Fig. 3g**.

2) An assertion is made in multiple places that "trimer-free patches" (line 189) are "devoid

of trimeric outer membrane proteins" (line 208). I think the authors mean "void", but my English might not be up to par. Nevertheless, the assertion is rather strong. These areas seem void of the porous network reminiscent of trimeric outer membrane proteins. You do not molecularly label these zones to know whether they are or are not protein-free, even though they may very well be. The colicin tag could solve this, but again that might be a technically challenging and time-consuming experiment. It may very well suffice to say that there are smooth patches void of the pore network reminiscent of trimeric outer membrane proteins. If there are or are not proteins located there that the authors may or may not be able to resolve by AFM does not retract the validity of the work in this manuscript.

We appreciate the comment regarding the assertion that the observed patch regions are free of protein trimers.

Our previously published work found that biochemically specific ("molecular") labels for (trimeric) OmpF and (monomeric) OmpA did not colocalise with the patch-like regions but did colocalise with the areas covered by pore networks (PNAS, 2021, 10.1073/pnas.2112237118). Based on the smooth/flat appearance of the patches and the lack of colocalization of some of the (in *E. coli)* most abundant trimeric and monomeric OMPs with the smooth patches, we there concluded that the patches are largely free ("devoid") of proteins. We have carried over that conclusion in this manuscript. Nonetheless, since the complete absence of proteins in the patch-like areas is not proven in this manuscript, we have now tempered the according assertion in **lines 214-217**.

3) Line 164, the authors aimed to resolve outer membrane porin structures reminiscent of protein trimers (OmpF and OmpC).

We thank Reviewer #2 for their phrasing suggestion and have changed the text accordingly in **lines 166-168**.

4) Line 179-181, sentence could use a reference.

We thank Reviewer #2 for their suggestion and have added the foundational paper (*Microbial cell factories*, 2015, 10.1186/s12934-015-0241-5) as the reference in **line 187**.

5) Paragraph 212-22: A bar chart or some other quantitative representation alongside the Western blot would be helpful to corroborate the claim made that OmpC levels are similar between clear and rough strains of E. coli. The difference in staining by Western blot for OmpF is apparent by eye; the difference or similarity in OmpC expression is slight, but if one corrects for the control band (which is not mentioned in the figure legends), it

could very well be that the difference in OmpC expression is statistically significant. This does not change the main conclusions of the manuscript, but the accuracy of the assertion made in the results is important.

We thank Reviewer #2 for their suggestions and have added the comparison of OmpC expression levels (relative intensity) to the supplementary information **Fig. S4**.

6) For Figure 3E, why not show phase images, which tend to have greater contrast than the height channel? Would we be able to resolve the absence of surface pores easier?

We thank Reviewer #2 for their comment and suggestion. Overall, we find that for observing pore features on *E. coli* by AFM, the height and phase images are most consistent, providing a basis for using whichever of the two that provides that the best contrast (PNAS, 2021, 10.1073/pnas.2112237118). We agree that phase images can provide impressive contrast, as also illustrated in **Fig. 3a** of this manuscript for rough and smooth MG1655 cells. However, the relative contrast observed in phase images depends on the imaging conditions and the features being studied. We found that phase images can provide superior contrast in the presence of the pore features associated with trimeric outer membrane protein assemblies. However, in the absence of these features, phase images do not always provide enhanced contrast or a clear advantage over height images. In response to this reviewer comment, we have now included the direct comparison of the height and phase images of the clear MG1655 cells in the supplementary information as **Fig. S3**. As shown in this new **Fig. S3**, phase images do not provide additional contrast for clear *E. coli* images.

7) For figure S3. Mention in the figure legend what the boil denotes. It is mentioned on line 557 that half of the sample was boiled presumably to liberate proteins from the outer membrane sample. I expect that the control band is at the bottom of each boiled lane in the Western blot. Mention in the figure legend that it is the control and mention the antibody used and protein labeled in the methods section. If there is no control, then I am afraid that comparisons between samples cannot be made, only bands within each sample. Were the blots only labeled with anti-OmpF? Is this a monoclonal or polyclonal antibody that also labels OmpC or is OmpC naturally labelled by this antibody. Was this antibody procured or harvested specifically for this study from rabbits, I presume?

We thank Reviewer #2 for highlighting missing information on the Westen blot methods in what is now **Fig. S4** in this revised submission. The antibody being used is Polyclonal anti-OmpF antibody (Biorbyt, #orb752372) which is cross-reactive with OmpC. We now have added details concerning the antibody used in **lines 616-617**. The control band at the bottom of the western blot in **Fig. S4a** has now been labelled “control” and represents the control used for intensity comparison between the bands.

8) On line 256 it is mentioned that glutaraldehyde exhibits a 2- to 5-fold higher number of cells immobilized of (A. baumannii and S. aureus). Where is the quantification of this observation?

We thank Reviewer #2 for highlighting the missing reference to **Fig. 4b** for this statement, which compares the immobilisation of the different bacterial strains on glutaraldehyde and Vectabond™ coated surfaces. We have now added a reference to this figure in **line 274**.

9) The authors mention on lines 461-462 that they used a 2% glutaraldehyde solution for functionalizing the coverslip surface. Is this optimal? Is it possible to quantitate a coating efficiency per surface area? Comparisons are made for how well glutaraldehyde functionalization enables bacterial immobilization. Some sort of quantification for how many bacteria (of specific strains or species) adsorb on the coverslip surface functionalized with specific concentrations of glutaraldehyde solution would be helpful for qualifying how impactful the comparisons are made by the authors. This is perhaps pertinent for understanding how and why rod-shaped bacilli can be found adherent in an upright orientation that is favorable to image polar regions (lines 318-322).

We thank Reviewer #2 for their questions regarding coating efficiency at varying glutaraldehyde concentrations. The attachment efficiency, quantified from bright-field images of bacteria immobilised on glass coverslips, is provided in the main text as cells per field of view (FOV), with the FOV dimensions specified in the Methods section (**lines 541-544**). To improve clarity, we have now added the FOV dimensions directly to the figure captions (**lines 114** and **315**).

In response to this reviewer comment, we performed additional experiments to assess the attachment efficiency and cell viability as a function of glutaraldehyde concentrations for *P. aeruginosa* PAO1, the bacterium we found least readily immobilised by charge-based functionalisation. We tested glutaraldehyde concentrations of 0, 0.5, 1, 2, 4, and 8%, immobilising 200 µL of exponentially growing PAO1 ($OD_{600}$ = 0.5) for 20 minutes. We found that glutaraldehyde concentrations between 1 and 8% provided stable attachment of *P. aeruginosa* over two hours, whereas cell attachment was significantly reduced at lower concentrations (0 and 0.5%) (**Fig. S7**), where the adhesive forces may predominantly be due to the underlying APTES coating, which provides a positive surface charge similar to Vectabond™.

Analysis of SYTOX™ positivity further indicated high membrane integrity for bacteria immobilised on 1-8% glutaraldehyde, whereas viability decreased significantly at lower concentrations (0 and 0.5%). Overall, we found that 2% glutaraldehyde provided sufficient attachment for all tested cell types during AFM measurements (**Fig. 2** and **Fig. 4**). While 2% glutaraldehyde was adequate for our experiments, we recommend that

researchers optimise the coating concentration according to the cell type under investigation and the intended experimental conditions and added this information to the discussion in **lines 417-422**. We have added the comparison of *P. aeruginosa* attachment on slides coated with different glutaraldehyde concentrations to the main text (**lines 270-272**) and included the corresponding figure in the supplementary information as **Fig. S7**.

10) Functionalization with lectins is intriguing as well for the fundamental biology that can be revealed for individual bacteria. However, lectins often become covered in gunk and their binding affinity becomes attenuated. I imagine that glutaraldehyde and perhaps even Vectabond also age at certain rates for these sorts of experiments. It is commendable that the authors show a quantification of bacterial immobilization after 2 hours. This may represent a few generations of the cell cycle for bacteria grown at their "optimal" lab culture conditions. Figure 2e provides some indication that cells can divide. This immobilization strategy is relevant for studying fundamental cell processes and features on a short-midterm timescale, which is commendable in itself. Is that the extent of it, or can this study already project further to studying more long-term timescale cell processes relevant to pathogens (though perhaps in non-pathogenic models) like stress tolerance?

We agree with Reviewer #2 that all surfaces will age over time especially with proteins in solution that could block the surfaces and prevent further cell attachment, with further enhanced risk of detachment any time cells divide. In the first instance, here we have explored if bacterial immobilisation is sufficiently durable to track bacterial growth and division, as also shown in **Fig. 2e**. For longer-term studies, many variables come into play, also including growth medium and temperature. We enthusiastically share this interest with the reviewer and expect to explore it in future work; we have added a comment on this outlook to the manuscript (**lines 442-444**).

11) The authors use SYTOX as a proxy for cell viability, as a commonly used indicator for "living" and "dead" cells. It is not a perfect tool as bacteria do at times stain positively with SYTOX and yet remain alive. Is there any understanding as to how many cells of the same cells are tracked and considered SYTOX positive or negative, exhibit growth or not, and are or are not observed to divide within a 2-hour window, within a field of view, in a time-lapse? This might not be technically possible in the authors' setup if there is not temperature control and thus the time required for a cell cycle at room temperature is longer than the 2-hour long studies done or the 33-min time-lapse show in figure 2e of an E. coli cell undergoing division. Another possibility would be to track how many cells are int eh process of dividing as often in bacteria septum formation (even incomplete) is detectable.

We agree with Reviewer #2 that SYTOX is not a direct measure of cell death and the inability for cells to grow and divide. SYTOX labels bacteria with compromised cell envelopes (*Applied and environmental microbiology*, 1997, 10.1128/aem.63.6.2421-2431.1997). Thus, our results could overestimate the quantity of dead cells in the immobilised population. We have therefore revised the manuscript in **lines 133-135** and **lines 115-116** to better clarify that SYTOX is used as an indicator of compromised membrane integrity rather than a definitive measure of cell death.

The reviewer raises the question if SYTOX ("dead") staining anticorrelates with the potential of cells to continue growing, which could indeed be addressed by tracking growth and division of individual cells. Because its relatively low throughput, AFM would not be the best method for these measurements. Instead, this would call for optical, single-cell tracking experiments as the spatial resolution will be sufficient to assess cell division of many cells in parallel over time. This is beyond the scope of this manuscript.

12) Line 128-129 brings up a point that washing removes unadhered cells. It is also important to note that bacteria even within isogenic, clonal populations are phenotypically heterogeneous to various extents. It could very well be that unadhered cells are non-adherent for mechanical reasons, that they never had their chance to adsorbed within 30 min of incubation (bad luck) or that their surface physiology does not make them amenable to imaging. This should not take away from the manuscript, as now we can at least envisage imaging cells, but perhaps not all and for biological reasons.

We agree with Reviewer #2's comment that our immobilisation approach may preferentially facilitate the attachment of certain bacteria within a population and, consequently, may not capture the full heterogeneity of the population. We have now highlighted this possibility in **lines 420-422** of the discussion.

13) In Figure 2b, attachment efficiency was quantified from brightfield images. Are these AFM-imageable bacteria? How would a quantification using brightfield compare to AFM imaging efficiency? At the end of the day, the manuscript cares about whether we can readily image adherent bacteria.

We thank Reviewer #2 for this question. While optical microscopy does not necessarily translate into AFM imageability, our experience shows that after repeated rinsing to remove non-adherent cells, optical microscopy is a good predictor for AFM imageability. We quantified cell attachment immediately after immobilization and again after two hours, including repeated surface rinsing. Poor attachment typically results in substantial cell loss after 2 hours and often translates into subsets of cells in the population not being suitable for AFM imaging from the beginning. Insufficient cell attachment, for instance, is highlighted in our response to Reviewer #2 point 9, where

substantial cell loss is recorded over two hours for low concentrations of glutaraldehyde (0, 0.5%). In contrast, when attachment remains stable over two hours, the optical microscopy images thus far have translated well into successful AFM imaging.

14) Phase images can offer impressive contrast as compared to height images. Is there a reason not to show phase images in figure 3e to potentially reveal the pore network in rough E. coli and its absence in the "clear" strain?

Due to the similarity to Point 6 raised by Reviewer #2 we refer to our answer in Point 6 and hope it addresses the Reviewer's point accordingly.

15) Figure 4d, mention in the figure legend (lines 301-302) that these images are of the height channel (I assume that is what they are). Or is this a 3D representation of height images?

We thank Reviewer #2 for highlighting the missing statement of the specific image type shown in **Fig. 4d**. **Line 321** now mentions that height images were displayed.

16) On lines 261-262 it is mentioned that "height undulations at the nanoscale" are observable, but I don't know what the authors refer to. Are they mentioning the seeming protrusions from the surface? An undulation is well defined in mechanics as having a rhythmic pattern of wavelength, amplitude and phase. A color bar is needed for Figure S5.

We thank Reviewer #2 for highlighting our unclear phrasing. We have rephrased the statement in **lines 279-281** to more clearly describe that *P. aeruginosa* shows height variations in the form of smaller and larger protrusions. We additionally added line profiles of these protrusions to **Fig. S8**. We further excuse the previous miss of adding a colour and scale bar to **Fig. S8**., now corrected.

17) Lines 316-319: is there data quantifying how discrete incubation times increase the rate of attachement of MG1655 in an orientation favorable to imaging polar regions?

We thank Reviewer #2 for their question. We have not further explored the effect that incubation time plays for more favourable polar attachment.

18) Line 335, an assertion is made that the morphology of a bacterial pole being hemispherical is an "ideal" shape and the pole of a cell harvested from exponential growth phase exhibited "morphological defects". What is the ideal nature of the pole shape referring to? From a biological perspective, an individual bacterium that the authors can image by AFM is in a certain physiological state in stationary or exponential

phase of bulk growth conditions, which may or may not be ideal or defective for any number of other reasons. E. coli has extracellular structures like fimbriae or flagella. There is no squiggle to indicate that these points in Figure 5c are long and in motion, but these "defects" might very well not be defects at all.

We thank Reviewer #2 for highlighting that our statement was unclear. We therefore have rephrased the statement to highlight the differences observed between bacteria in exponential and stationary growth phase in **lines 355-357**.

19) Line 369, Figures 2 and 4 are cited for presenting evidence of cell viability by SYTOX staining: okay. On line 368 it is mentioned that cells are observed to be able to grow and divide. I see evidence of division in figure 2e, though not really growth (unless a kymograph can be produced or a quantification of elongation conducted). Furthermore, I do not see where there is evidence that the other bacterial model organisms are observed to grow and divide. We at least have evidence for "viability" as measured by SYTOX and division for E. coli.

We thank Reviewer #2 for highlighting that our statement was too general and as such not supported by the provided data.

**Line 389** was rephrased to more accurately describe our observation that specifically rough *E. coli* was able to grow as evidenced by the clear elongation of the cell between 0-24 min in **Fig. 2e** followed by cell division. To highlight the specific time frames showing growth and division we further mention them directly in the main text **lines 162-163**.

20) Lines 393-395: it is mentioned that AFM imaging of S. aureus revealed "peptidoglycan architectures as reported previously". Again, these are likely peptidoglycan architectures, but there is not a molecular labeling of these architectural structures to indicate that there are composed of peptidoglycan. They are reminiscent of peptidoglycan structures reported previously.

We thank Reviewer #2 for their suggestion and have rephrased the sentence in **lines 415-416** accordingly.
